# Fossils and living taxa agree on patterns of body mass evolution: a case study with Afrotheria

**DOI:** 10.1098/rspb.2015.2023

**Published:** 2015-12-22

**Authors:** Mark N. Puttick, Gavin H. Thomas

**Affiliations:** 1 School of Earth Sciences, University of Bristol, Wills Memorial Building, Queen's Road, Bristol BS8 1RJ, UK; 2 Department of Animal and Plant Sciences, University of Sheffield, Alfred Denny Building, Western Bank, Sheffield S10 2TN, UK

**Keywords:** evolution, fossil, body mass, ancestral size reconstruction, Afrotheria, macroevolution

## Abstract

Most of life is extinct, so incorporating some fossil evidence into analyses of macroevolution is typically seen as necessary to understand the diversification of life and patterns of morphological evolution. Here we test the effects of inclusion of fossils in a study of the body size evolution of afrotherian mammals, a clade that includes the elephants, sea cows and elephant shrews. We find that the inclusion of fossil tips has little impact on analyses of body mass evolution; from a small ancestral size (approx. 100 g), there is a shift in rate and an increase in mass leading to the larger-bodied Paenungulata and Tubulidentata, regardless of whether fossils are included or excluded from analyses. For Afrotheria, the inclusion of fossils and morphological character data affect phylogenetic topology, but these differences have little impact upon patterns of body mass evolution and these body mass evolutionary patterns are consistent with the fossil record. The largest differences between our analyses result from the evolutionary model, not the addition of fossils. For some clades, extant-only analyses may be reliable to reconstruct body mass evolution, but the addition of fossils and careful model selection is likely to increase confidence and accuracy of reconstructed macroevolutionary patterns.

## 1. Introduction

Body mass evolution of Mammalia has received considerable attention in the literature [1–11]. Particular interest has been shown in changes in body size following the K–Pg mass extinction [1], modes of evolution [2,5] and how rates vary through geological time [3,7]. Many studies have approached these issues from an extant-species-only perspective (e.g. [3,4]), but there is an increasing awareness of the importance of including fossils in macroevolutionary analyses [5,6,8–10,12].

Studying events in deep time using only extant taxa is problematic, as ignoring fossil data can introduce biases and inaccurate reconstruction of phylogenies and macroevolutionary patterns [13]. Furthermore, when studying morphological change, the inclusion of fossils can improve ancestral state estimates in deep time: models with fossil information may fit better than models without [5,6,8–10], and fossil evidence can be used as prior information on ancestral body mass [8]. However, there is some suggestion that studies of macroevolution may be obscured by fossil evidence as it can obfuscate patterns by introducing its own biases [11]. One area that is particularly sensitive to the inclusion of fossils is ancestral state reconstruction. Ancestral state reconstruction is generally difficult [14,15] and ignoring fossil evidence can lead to over-inflated estimates of ancestral mass [6].

Methodological approaches, as well as the inclusion of fossils, can greatly influence interpretations of macroevolution. Many methods use a gradualistic Brownian motion (BM) model to study body mass evolution [16–19], and many approaches have built on this framework to study evolutionary tempo [3,20,21] and mode [17–19,22,23]. Recently, parametric approaches have been used that can model gradual evolution with sporadic bursts [24,25], so these are not rooted in the gradual evolution expectation of the BM model. Currently, the relative influence of model selection versus the inclusion or exclusion of fossils on our understanding of evolution is unclear. Indeed, it may be that models and fossils matter crucially in some circumstances, but not in others.

A first step to understanding the relative impacts of fossils and models on ancestral state reconstruction is to reconcile extant (typically molecular) and fossil (morphological) phylogenies. Recently developed methods allow for the incorporation of living and fossil data in phylogenies, by enabling the concurrent analysis of molecular and morphological characters [26,27]. An important step in this process is the use of fossils as tips to date phylogenies [26,27] compared with traditional node dating. Total-evidence dating resolves previous problems of uncertain assignment of fossils to nodes by including fossils in the phylogenetic analysis [28] and it has also been suggested that molecular data improve the resolution of phylogenies containing fossils [29].

Here we test the influence of the inclusion and exclusion of fossils on the rates and modes of afrotherian body mass evolution. Using a total-evidence analysis [27], fossils were incorporated from a morphological matrix [30], and evolutionary models were compared with both a traditional molecular-only node-dated tree, and a total-evidence tree that had the fossils removed.

Afrotheria, which includes elephants, hyraxes and tenrecs, consists of approximately 77 extant species [31–33]. The general consensus on their relationships is that Afrotheria comprises two clades: Afroinsectiphilia, including Tubulidentata (aardvark), Afrosoricida (Chrysochloridae plus Tenrecidae) and Macroscelidea (elephant shrews), and the generally larger-bodied Paenungulata, including elephants and hyraxes [30,33]. Fossil afrotheres are known throughout the Cenozoic [34], and living forms are known to have a wide variation of body size that spans six orders of magnitude.

Surprisingly, we find the inclusion or exclusion of fossil tips has little impact on analyses of body mass macroevolution: with all phylogenies there is a relatively small ancestral body size for Afrotheria, and a branch-based shift in rate leading to Paenungulata and Tubulidentata. No datasets support BM models of evolution, and parametric rate-variable approaches indicate a smaller ancestral mass compared with BM estimates. The addition of fossil tips on the phylogeny here has little impact on evolutionary rate analyses, but there are differences attributable to model selection. While inclusion of morphological characters and fossil species alters phylogenetic topology, these differences result in negligible differences in patterns of body mass evolution or ancestral body mass estimation. In some cases of macroevolutionary analyses, as here, it may be possible to reconstruct evolutionary history while using extant species only, although the addition of fossils will increase confidence of reconstructed patterns.

## 2. Material and methods

### (a) Taxa

We recognize a total of 77 extant afrotherian species (see electronic supplementary material, S1) [31], and we used a morphological matrix of fossil and extant afrotheres [30,35]. The matrix contains a sample of fossil taxa across Afrotheria, and these fossils are generally early-diverging members of crown clades, so it is likely that they give good estimates of ancestral morphology and timing of diversification [27,35]. We sample a total of 39 afrotherian fossils based on morphological data only and a further seven taxa for which molecular data are available (see below). For Afrotheria, the morphological data sample all extant orders, as well as fossil members of extant orders. Within Afrotheria, these fossil taxa are believed to be stem or crown members of extant families, with the possible exception of *Chambius kasserinensis* and *Herodotius pattersoni* [35]. Extant outgroup taxa were selected from Xenathra (three species), Boreoeutheria (13 species) and marsupials (three species). Additionally, we sampled two fossil crown placentals (*Montanalestes keeblerorum* and *Prokennalestes trofimovi*; see electronic supplementary material, S1).

### (b) Genetic data

Genetic data were taken for six nuclear and four mitochondrial loci from GenBank [33,36]. Genetic data were aligned using ClustalW [37], with protein-coding genes aligned by codons and non-protein genes by nucleotide. Unalignable regions were removed from non-coding sequences using GBlocks (v. 0.91b) [38].

The following genes were used in the analyses: growth hormone receptor (GHR), alpha-2B adrenergic receptor (ADRA2B), androgen receptor (AR), von willebrand factor (vWF), interphotoreceptor retinoid-binding protein (IRBP) and brain-derived neurotrophic factor (BDNF) were the nuclear protein-coding genes, and cytochrome *b* (cyt*b*) and nicotinamide adenine dinucleotide (NADH2) were the two mitochondrial protein-coding genes. Additionally, sequence data from the mitochondrial 12 s and 16 s genes were collected. The dataset differs from Kuntner *et al*. [33] by the addition to BDNF and some additional data for some species (see electronic supplementary material, S1). Of the 77 extant species recognized, we have genetic data for 60 (approx. 78% of the total). When extinct species that have genetic information are included, coverage for Afrotheria species ranges from 67% for GHR to 25% for AR.

Data were also collected for extinct species in the analysis. As with Kuntner *et al*. [33], we gathered information on the proboscideans *Elephas antiquus falconeri*, *Elephas cypriotes*, *Elephas maximus asurus* and *Elephas* sp., and an undetermined species from Tilos island [32]. We also included the mastodon *Mammut americanum,* the mammoths *Mammuthus primigenius* and *Mammuthus columbii*, and Steller's sea cow (*Hydrodamalis gigas*).

All alignments were checked by eye. PartitionFinder (v. 1.1.1) [39] was used to select the partitions of genes and models of evolution for the genetic data. For most genes the best-fitting substitution model was the general time-reversible (GTR) model with gamma distributed rate variation between sites and a proportion of invariant sites. Exceptions to this model were the GTR with gamma-distributed rate variation and no invariant sites (cytB), the Kimura 82 model (GHR) and the Kimura 82 model with a proportion of invariant sites (BDNF).

### (c) Phylogenies

Phylogenies were constructed and dated in MrBayes v. 3.2.5 [40]. All phylogenetic analyses were run for 20 million generations, sampling every 1000 generations, with four chains and four independent runs for each analysis. The heating parameter was set to 0.05 for analyses that included fossils and 0.1 for analyses that did not include fossils. Priors were set using established protocols [27] (see electronic supplementary material, S1), and convergence was judged using in-built diagnostics of MrBayes and Tracer [41].

An initial non-clock analysis was run on the entire dataset of fossils and extant species, with no calibration on ages (see electronic supplementary material, S5 and figure S2).

### (d) Time-calibrated analyses

We conducted three sets of dating analyses: (i) node and tip dating using both morphological and molecular data (total-evidence analysis), (ii) node-only dating using molecular data only (node-dating analyses), and (iii) node-only dating using both morphological and molecular data. For both the total-evidence and node-dating analyses, the following nodes were calibrated at Theria (root), Marsupialia, Placentalia (crown), Boreoeutheria, Atlantogenata, Xenarthra, Afrotheria, Paenungulata and Macroscelidea. Node dates were set as offset-exponential distributions with dates primarily taken from a published source [42]. For the total-evidence analysis, tip dates came from 41 unconstrained species believed to be Afrotheria and from two stem placentals. Tip dates for fossils were set as uniform distributions, with dates taken from the FossilWorks [43] portal, which accesses data in the Paleobiology Database [44] (see electronic supplementary material, S6). However, these data were further checked using the primary literature (see electronic supplementary material, table S2). For the total-evidence analyses, there were 50 dating points on the phylogeny (41 tips dates and nine node dates). In MrBayes, we set the fossilized birth–death model [45] as tree prior. The fossilized birth–death model relaxes the assumption of a uniform prior between the timing of nodes and incorporates estimates of speciation, extinction and fossil sampling rates into the tree prior. In this model, we assumed that fossil tips are sampled as branching lineages (‘Samplestrat = fossiltip’) but not as direct ancestors sitting on branches as is used in some models (i.e. not in the implementation in [46]). Priors for the speciation, extinction and sampling rates were set at their defaults as according to MrBayes v. 3.2.5: the speciation rate prior (‘SpeciationPr’) was set to an exponential distribution with rate 1, and the relative extinction rate prior (‘Extinctionpr’) and the relative fossilization rate (‘FossilizationPr’) were both set to a beta distribution (mean = 1, shape = 1) which gives a uniform prior between 0 and 1.

For the total-evidence analysis, the following topological constraints were applied: Marsupalia, Boreoeutheria, Atlantogenata, Xenarthra, crown Placentalia, Afrotheria, Paenungulata, Proboscidea, Sirenia, Hyracoidea, Macroscelidea, crown Macroscelidea and Chrysochchloridae. These clade memberships were based upon an initial unconstrained non-clock phylogenetic analysis.

### (e) Body mass data

Measurements of body mass were obtained for extant and extinct species in the phylogeny. Body mass data for extant species were predominantly taken from published estimates (see electronic supplementary material, S12). For the extinct species, the preferred data sources were from previously published mass estimates; when published data were not available, body masses were mainly estimated from regression equations on molar area [47] (see electronic supplementary material, S12).

### (f) Models of body mass evolution

Models of body mass evolution were tested on a selection of trees to assess the impact of fossils. For a direct comparison of the effects of fossils, body mass evolution was tested on the total-evidence phylogeny (i), and on the total-evidence phylogeny with fossils removed (ii). Furthermore, models were tested on the molecular-only node-dated phylogeny (iii), as this reflects the classic approach to construct time-calibrated phylogenies for comparative analyses. Additionally, models were tested on the node-dated phylogeny constructed using molecular and morphological data (iv).

The BM model is commonly used either to model trait evolution on phylogenies directly or as a basis for more complex models. The BM model assumes, on a phylogeny with branch lengths scaled to time, that variation in trait data accumulate proportionally through time, with a mean expectation of zero change in the value of the trait per unit time. However, the model makes assumptions that may be unrealistic [24,25]. The nature of the model means that variance is finite, and therefore rates are also finite and do not change in the phylogeny [25]. Therefore, to incorporate any rate variation the model must be extended with extra parameters to model changes in rate [3,20,21,48]. If this is performed over the entire phylogeny with each branch permitted to take a unique rate [48], the result is that the model has too many parameters for justifiable inference—a new rate on every branch in a fully bifurcating phylogeny results in nearly as twice as many parameters (2*n*−2, where *n* is tips) as data points (values at the tips). An alternative to modelling specific changes in rates is to use parametric models that do not assume constant rates, by sampling rates from a heavy-tailed, rather than normal, distribution [24,25]. This achieves two objectives: these models do not require a homogeneous gradual model of evolution, and they allow for an ancestral trait reconstruction with a model of rate evolution that is not over-parametrized.

We use the software StableTraits to parametrically model gradual evolution with intermittent bursts and to reconstruct ancestral size estimates and model rates through time [25]. StableTraits samples from a symmetrical, mean zero distribution which is defined by its index of stability (*α*): for BM *α* = 2, which results in a normal distribution, but when *α* < 2 this results in a shallower distribution with heavy tails, which allows for a more unpredictable evolutionary trajectory. For all trees, results from a heavy-tailed distribution in which the *α* is allowed to vary from BM were compared with a BM model in terms of the rates through time, ancestral size estimation and the model fit [25]. The MCMC chain was run for 2 000 000 iterations with four runs, until the potential scale reduction factor went below 1.01. The burn-in was set to 10%, with the output containing the calculated rates, ancestral states and maximum posterior probability. The model was tested against a model fixed to BM by re-running the analyses with *α* = 2, and then comparing the Bayesian predictive information criterion (BPIC) [25]. Subsequent data processing and plotting were carried out in R [49].

### (g) Prior information on ancestral mass

To introduce further information for the ancestral mass estimation for Afrotheria, an arbitrary outgroup tip was added and set a given mass to represent knowledge from the fossil record or ancestral estimates from previous studies; this outgroup was separated from Afrotheria by 5 Myr (the edge leading to the tip of the outgroup was 0.01 Myr); 5 Myr was the original length separating the Afrotheria from the Xenarthra and would allow prior information to influence the root, but the mass value could change over the length. In different analyses, the outgroup was given a mass of 0.1, 0.5, 1, 5, 10 and 20 kg. The values incorporate estimates for Late Cretaceous mammals from the fossil record (approximately 80 g [1]) as well as larger estimates for ancestral Afrotheria from genomic studies (approximately 0.5–30 kg; e.g. [4]).

## 3. Results

### (a) Topology and divergence times

**Figure 1.**
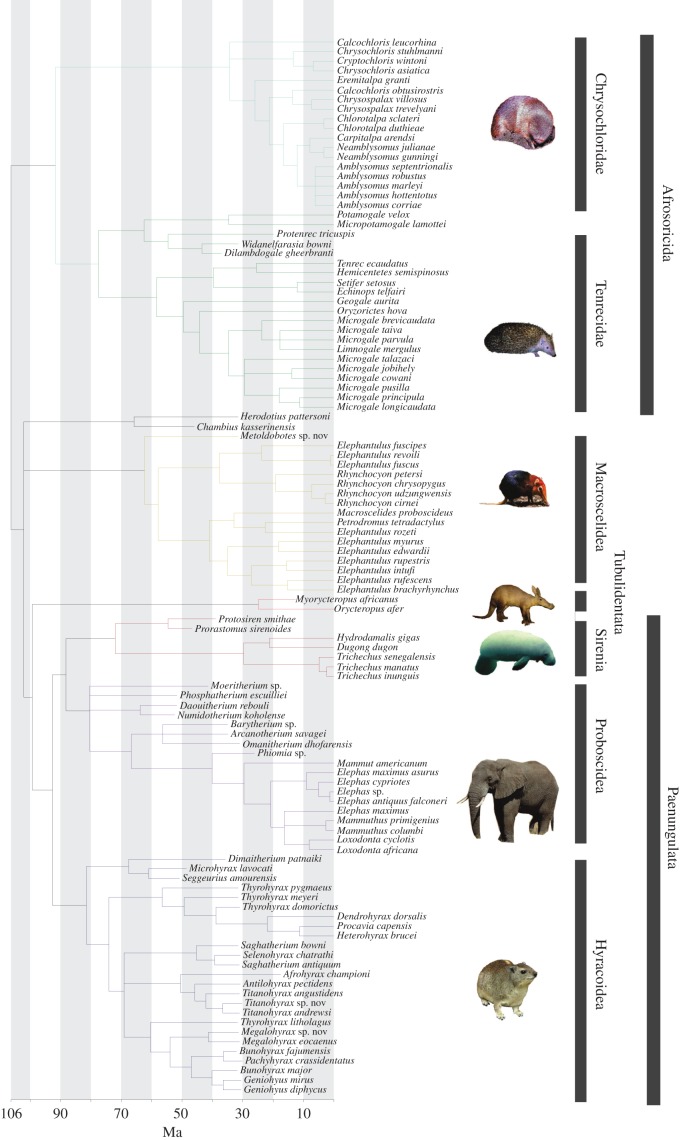
The dated total-evidence phylogeny indicates a late Cretaceous origin for Afrotheria. Tubulidentata and Macroscelidea form successive outgroups to the Paenungulata (Sirenia, Proboscidea, Hyracoidea), and so the Afroinsectiphilia (Macroscelidea, Afrosoricida) is non-monophyletic. All major clades are highlighted: Proboscidea (purple), Sirenia (brown), Hyracoidea (navy), Tubulidentata (red), Macroscelidea (yellow), Chrysochloridae (blue) and Tenrecidae (green). Animal images. The manatee image is public domain, and the others are from Wikipedia, covered by Creative Commons licences that are attributed to the following authors: elephant (Ikiwaner), hyrax (D. Gordon E. Robertson), aardvark (Masur), elephant shrew (Joey Makalintal), golden mole (Hohum) and tenrec (Wilfried Berns).

The total-evidence phylogeny (figure 1) and non-clock phylogeny (see electronic supplementary material, figure S2) are very similar, and the composition of all the major clades is identical.

Larger differences are seen when morphological data are included compared with molecular-only topologies: in all analyses with the morphological cladistic matrix Afroinsectiphilia is not monophyletic as Macroscelidea is closer to Paenungulata. The composition of crown families is consistent, but the position of fossil taxa does vary between analyses. For example, the fossils *Chambius* and *Herodotius* move from sister of Paenungulata plus Tubulidentata in the non-clock topology to being in a basal polytomy with Macroscelidea in the total-evidence analysis.

Ages from the total-evidence analysis that includes fossils (figure 1) are older than the ages from node-dating analysis (table 1).

**Table 1.** Dates from the total-evidence analyses are older than the node-dating analysis but the 95% posterior density shows overlap for crown Afrotheria.

	total evidence	node dating (molecular and morphological data)	node dating (molecular only)
Afrotheria	106.3 (91.3, 123.9)	96.7 (78.6, 116.9)	92.9 (74.3, 114.5)
Paenungulata	99.3 (85.3, 115.4)	61.5 (55, 74.8)	61.8 (55, 76.2)
Afroinsectiphilia	n.a.	n.a.	90.2 (71.6, 110.7)
Proboscidea	29.5 (18.9, 41.1)	23.9 (14.6, 33.8)	24.5 (15.3, 34.5)
Sirenia	29.6 (17.6, 43.9)	26.9 (15.9, 39.7)	27.3 (15.5, 39.4)
Hyracoidea	21.9 (12.1, 33.3)	18.5 (8.4, 29.1)	18.7 (8.4, 28.8)
Afrosoricida	91.6 (77.1, 109.0)	89.5 (71.5, 109.1)	85.1 (67.3, 106.1)
Tenrecidae	77.5 (62.2, 92.2)	78.8 (62.0, 97.8)	76.7 (59.5, 96.3)
Chrysochloridae	34.3 (23.4, 46.7)	39.6 (27.6, 53.3)	40.4 (27.8, 56.0)
Macroscelidea	57.8 (45.0, 71.9)	75.2 (58.0, 95.7)	77.5 (58.6, 98.4)

### (b) Ancestral states

For all analyses, neither rates nor ancestral body size reconstructions are strongly influenced by the inclusion of in-group fossils. Additionally, for all analyses the StableTraits model provided a better fit for the data than BM.

**Figure 2.**
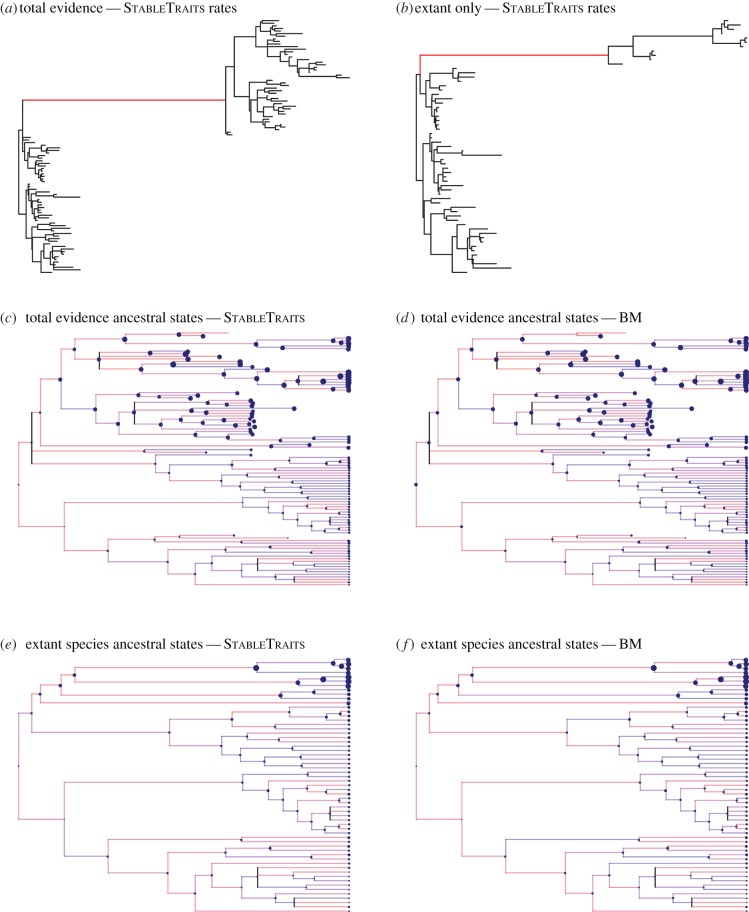
The effects of model selection are more evident than the inclusion of fossils. (*a,b*) If fossils are included or excluded, there is a large increase in the morphological rate of evolution leading to the Paenungulata plus Tubulidentata (red branch). The reconstructed body size is comparable between the total-evidence and neontological studies that use (*c,d*) the StableTraits models and (*e,f*) the BM model.

In the total-evidence approach with no outgroups and rate-heterogeneous (StableTraits) model the ancestral size at the origin of the Afrotheria is estimated to be 0.10 kg (95% CIs, 0.02–0.95 kg). By contrast, the BM estimate is an order of magnitude larger 1.45 kg (95% CIs, 0.31–6.82 kg); however, the broad confidence intervals overlap with those of the rate-heterogeneous model (table 2 and figure 2). The fit of the heavy-tailed rate-heterogeneous model (*α* = 1.77, 1.47–1.94) was superior to the BM model (*α* = 2; ΔBPIC = 21.8).

**Table 2.** Reconstruction of ancestral body size using StableTraits indicates the minimal impact of fossil tips on root mass estimates.

phylogeny	StableTraits	Brownian motion	best-fitting model	ΔBPIC
total evidence	0.10 (0.02, 0.95)	1.45 (0.31, 6.82)	StableTraits	21.76725
extant only	0.13 (0.02, 12.48)	1.59 (0.28, 8.94)	StableTraits	53.5555
node dating (molecular only)	0.11 (0.02, 761.4)	0.77 (0.14, 3.99)	StableTraits	57.42925
node dating (molecular and morphological data)	0.09 (0.02, 0.62)	0.53 (0.10, 2.75)	StableTraits	42.499

Removal of fossils caused little difference in the ancestral size estimation of Afrotheria (0.13 kg), but had a marked effect on the confidence intervals, which became much wider (0.02–12.48 kg). For the molecular-only node-dating analysis, the ancestral size estimate for Afrotheria was 0.11 kg (95% CIs, 0.02–761.4 kg). Similar results were found for the combined morphological–molecular node-dating analysis (table 2).

### (c) Evolutionary rates from stabletraits

In all StableTraits analyses, there is an increase in the rate of body mass evolution leading to the Tubulidentata plus Paenungulata (figure 2). For the total-evidence analysis, the increase leading to Tubulidentata plus Paenungulata is 137.7 times the original branch length (length of the identical branch on the time-scaled input phylogeny; figure 2), compared with an increase of 117.0 times the original length when fossils are removed from the phylogeny. The rate increases are less dramatic for the molecular-only node-dated phylogeny (35.2 times the original rate) and the morphology and molecular node-dated phylogeny (19.9 times the original rate). On the morphology and molecular node-dated tree with only extant taxa there is also a further increase (37.3 times the original rate) leading to the Proboscidea plus Sirenia.

### (d) Impact of prior information

The addition of outgroups of variable mass (0.1 to 20 kg) had little impact on estimates of ancestral mass for Afrotheria (electronic supplementary material, table S2 and S3) or rates through time (electronic supplementary material, figure S7). Even when the outgroup represents a body mass that is much larger than those known from the fossil record (e.g. 20 kg), the mass estimates from ancestral Afrotheria are relatively small (approx. 2 kg), indicating the stability of the reconstructed patterns in this study.

## 4. Discussion

Congruent patterns of body mass evolution are produced when fossil tips are included or excluded. The addition of fossil tips to analyses has little effect on the analyses of ancestral mass estimation and rates of body mass evolution through time. A number of studies have argued that fossils are vital to understand patterns of body mass evolution [6,8–10], but results from analyses in Afrotheria are consistent if fossil tips are included or excluded from phylogenies. The minor impact of fossil tips on macroevolutionary interpretations in this case may be expected: the afrotherian fossil record is biased towards Paenungulata [34], and none of the fossils in the clades is larger or smaller than extant members of those clades. Furthermore, there is generally a bias in the fossil record of the two groups: with the exception of Macroscelidea, the fossil record of Afroinsectiphilia is not as comprehensive as the record of Paenungulata [34], but there is fossil representation of all the major clades included in our analyses. There is no evidence to suggest that earlier afroinsectiphilians (excluding tubulidentates) were much larger than today's species, whereas some extinct hyraxes were indeed much larger than their extant relatives. Fossils, or at least morphological character data, do have large impacts on the topology of Afrotherian phylogeny. However, these differences in topology do not have a large impact on analyses of body mass evolution in this study, but instead show how different data types and fossil inclusion can change our interpretations of evolution. More evident than the inclusion or exclusion of fossils is the impacts of model selection.

Despite the minor impact of fossils in estimating ancestral body size in the Afrotheria, we do not suggest that these results should be taken as grounds to ignore fossil data. Previous studies have demonstrated the need for phylogenetically informed sampling for ancestral state reconstruction [50]. Recent studies have suggested the results here—that fossils have little impact upon reconstructions of morphological evolution—may not be applicable to other clades, such as birds [51], or even all mammals [6,8–10]. As noted above, the distribution of fossil tips and sizes may explain their minor impact in this specific case. The omission or misplacement of taxa, whether fossil or extant, can affect estimates of evolutionary rates and ancestral states. Moreover, our results suggest that inclusion of fossil data may increase confidence in ancestral state estimates. Fossils may still be very important in studies of body mass evolution, but exploration of alternative evolutionary models can also be important. A recent study has shown that careful model selection can elucidate body mass evolution patterns from extant data that have previously only been shown in fossils [52]; here we support that the evolutionary model can have a large impact on our interpretations of evolution. It will often be difficult to judge *a priori* whether fossils or the evolutionary model will matter more and as such both should be assessed wherever possible.

The largest difference in reconstructions of body mass evolution in Afrotheria is not when fossils are included or excluded, but when comparing alternative evolutionary models. Mesozoic mammals, including early Placentalia, have been shown to be generally small (approx. 80 g) [1] and high morphological rates of change are found early in the evolution of clades [7]. By contrast, genomic studies have indicated a larger ancestral mass for Afrotheria [4,53]. Our results are congruent with the fossil record, whether fossils are included or excluded (figure 2). Furthermore, other studies have found similarly small ancestral sizes for the Afrotheria (0.36 kg) using the same method (StableTraits) but different data [25]. There is approximately 10-fold difference in estimates from StableTraits and BM (table 2); this suggests that model selection, rather than inclusion of fossils, has a greater impact in reconstructed ancestral body mass. However, it should be noted that in all cases the confidence intervals for StableTraits and BM ancestral size estimates overlap (table 2). While there are general difficulties in reconstructing ancestral mass [14–15], fossil tips do not necessarily impact on either the best-fitting evolutionary model or the ancestral state estimates. Our results appear to be robust to the possibility of undiscovered afrotherian species with extreme body sizes as demonstrated by the very minor effect of manipulating a proxy prior on the root. The main effect of an informed prior, such as previous estimates have shown (e.g. [4]), is to tighten the confidence intervals for ancestral state estimates.

Previously, total-evidence data have been shown to produce both younger and older ages than node dating [27,54], but other studies (e.g. [55]) are congruent with the results here in that the majority of node ages are older in the total-evidence analyses (see electronic supplementary material, figure S5). Here the evidence strongly suggests that fossils are pushing median dates back in time; a similar result has been found generally for all mammals [55]. While these ages are larger than large-scale molecular estimates [56], they are not implausible [55] and there is still an overlap in the posterior distributions of ages on the major nodes and root; thus there is no significant effect from the morphological matrix on divergence time estimation. Additional studies that have used the fossilized birth–death model [46] have found that using a method that allows for sampling fossils as direct ancestors generally results in age estimates that are more congruent with the fossil record [46,57–59]. However, many of these studies (e.g. [57]) find that traditional node constraints can result in ages that are congruent with the fossil record, which appears to be the case here.

## 5. Conclusion

Fossils have a vital role to play in the understanding of macroevolution. However, it is important to note that the addition of fossils will not always produce results that contradict analyses based on extant taxa. Data from fossils, in some cases, will agree with data from living species, so other factors, such as the choice of evolutionary model, are also likely to be important when elucidating patterns of evolution. Therefore, it may be possible to trust analyses based on extant taxa only, but incorporating fossil information and careful model selection can increase confidence in our interpretations.

## Supplementary Material

Supplementary Information

## Supplementary Material

InputFilesPhylogeny.txt

## Data accessibility

Input files for phylogenetic analysis and the body mass data are available in the electronic supplementary material.
