# Supplementary material for: Fossils and living taxa agree on patterns of body mass evolution: a case study with Afrotheria
Source: Proc Biol Sci. 2015 Dec 22;282(1821):20152023. doi: 10.1098/rspb.2015.2023 (PMC4707753; doi:10.1098/rspb.2015.2023)
Supplement: Supplementary Information [file rspb20152023supp1.docx]

**Supplementary information**

Fossils and living taxa agree on patterns of body mass evolution: a case study with Afrotheria

Mark N. Puttick^a^, Gavin H. Thomas^b^

^a^School of Earth Sciences, Wills Memorial Building, Queen’s Road, University of Bristol, Bristol, BS8 1RJ, UK

^b^Department of Animal and Plant Sciences, Alfred Denny Building, University of Sheffield, Western Bank, Sheffield, S10 2TN, UK

Table of contents

**Supplementary Methods…………………...………………………………….…………..2-6** 1. Taxa…………………………….…………………………………………………...2

2. Morphological clock model…..…………………………………………………..2-3

3. Phylogenetic priors………..……………………………………………………...3-5

4. Convergence…………….…………………………………………………………..5

5. Phylogenetic constraints...………………………………………………………......5

6. Temporal constraints…...………………………………………………………...…6

**Supplementary Results………………………………………………………...…….…...7-25**

7. Non-clock phylogeny……………………………………………….…………...7-12

8. Total-evidence phylogeny……..……………………………………..………...13-14

9. Node-dating phylogeny………………………………………………..……….15-17

10. Comparison of total-evidence and molecular ages………………...……………..18

11. Effects of prior information on ancestral state estimation ..………………….….19

12. Supplementary references………………………………………………...…..20-25

**Fossil Data………………………………………………………………………………..26-36**

13. Measurements and sources……………………………………………………26-28

13. Fossil ages……………………..………………………….………………..…29-30

14. Data references……………………………………………..……………........31-36

1. **Taxa**

Valid Afrotheria extant species were including from a compendium of mammal species [1], and more recent sources (for species described after the publication of the book): *Microgale jobihely* [2], *Rhynchocyon udzungwensis* [3]. *Oryzorictes talpoides* was considered as synonymous with *Oryzorictes hova*, and *Amblysomus* *iris* as synonymous with *Amblysomus* *hottentotus* [1]. When sourced from GenBank, *Huetia leucorhina* was used as a source for the species *Calcochloris leucorhina* as they are synonyms [1,4].

Outgroups were selected to represent a range of potential outgroups for the Afrotheria [5]. As the recognised inclusive clade is the Atlantogenata (Afrotheria and Xenarthra), the xenarthran taxa *Tamandua tetradactyla, Choloepus didactylus*, and *Dasypus novemcinctus* were selected as outgroups. Furthermore, taxa were included from a wide range of placental mammal groups: Scandentia (*Tupaia glis*), Eulipotyphla (*Condylura cristata* and *Erinaceus europeus*), Primates (*Homo sapiens* and *Lemur catta*), Rodentia (*Mus musculus*), Artiodactyla (*Sus scrofa*), Perissodactyla (*Dicernos bisornis, Equus caballus*, and *Tapirus indicus*), Pholidota (*Manis tetradactyla*), extinct early-diverging placentals (*Montanalestes keeblerorum* and *Prokennalestes* *trofimovi*) and Metatheria (*Macropus rufus, Phascolarctos cinereus,* and *Didelphis virginiana*). *T. glis, L. catta, E. europeus, D. virginiana, D. novemcinctus,* and *C. cristata* are represented by morphological and molecular data, and the early-diverging placentals *M. keeblerorum* and *P. trofimovi* are based on morphological data only; all other outgroup taxa are based on molecular data alone.

For Afrotheria, the morphological data samples all extant orders, as well as the fossil ancestors of extant orders [6]. Fossil sampling is more substantial in the Paenungulata compared to the Afroinsectiphilia [7]. For the morphological data [6,8], completeness, in terms of percentage of characters scored, ranged between 14% and 76% for fossil species in the matrix, with a mean of 45%. Character completeness for extant species ranged between 56% and 95%, with a mean of 85%.

1. **Morphological clock model**

Morphological data was set to the Mk substitution model [9] set to score only variable characters with gamma distribution between states. The Mk clock can be thought of as a form of the Jukes-Cantor 1969 nucleotide model of evolution, the differences being that in the morphological clock there are *k* states where *k* varies among characters, rather than four states that applies to all nucleotide data sets. As a Markov process along each branch, a change is allowed in any state at any point of time, but with no direction (no plesiomorphic or apomorphic states) [9]. So, changes in morphological states are independent, symmetrical, and the estimated branch length is equal to the number of changes between states in a time period [9]. In an extension to the model, rate heterogeneity between states is modelled as a gamma-distributed model [10]. Unlike in sequence data, where an ‘A’ nucleotide is the same in different genes or partitions, in morphological data a 0 designation is not the same in each character [9]. In the morphological model, a potential issue is that morphological data may only include variable data; characters states are never the same for all taxa, a problem known as acquisition bias, which leads to artificially extended branch lengths [9]. As a solution, the likelihood is calculated conditioned on there *only* being variable characters in the dataset [9. 10].

1. **Phylogenetic priors**

As with Ronquist *et al.* [10], an initial analysis was run as a non-clock model using extant species only to ensure that a sensible topology could be found and could be used as a scaffold for future analyses. In this case ‘extant’ is extended to include species for which molecular data is available, even if they are now extinct. A non-clock model was also run on the entire dataset of extant and extinct species.

When the non-clock initial tree was obtained, two analyses were run on this topology. The first of these was another non-clock analysis to obtain substitution branch lengths, and a separate strict-clock analysis on the same topology to obtain timed branch lengths. MrBayes only allows for a single taxa to be an outgroup [11], so the outgroup in the initial analysis was *Didelphis virginiana,* rather than all three Metatherian species found in the phylogeny. As a consequence there was a polytomy between the outgroup *D. virginiana* and the clade of *Macropus rufus* and *Phascolarctos* *cinereus.* When the subsequent non-clock branch lengths were estimated on this fixed tree, this relationship was unresolved, but for the strict clock tree the relationship was resolved with *D. virginiana* as sister to *M. rufus* and *P. cinereus*.

IGR model prior

The selection of priors for the clock rate and hyper-prior for the IGR model followed the protocol of Ronquist *et al.* [10]. The relaxed clock IGR model was used for analysis; this is an uncorrelated model that samples rates from a gamma distribution, and an expectation is that variance increases proportionally with time [10, 12].

Using estimates of non-clock and strict clock branch lengths a regression was performed. As in Ronquist *et al.* [10], the ratios between the non-clock and strict clock branch lengths were calculated; a regression between these values showed the expected variation around 1:1 (with an outlier); the outlier is the branch leading to the Eutheria. As the IGR is an uncorrelated model that samples rates from a gamma distribution, an expectation is that variance increases proportionally with time [10, 12]; a second regression of the squared deviation of the non-clock model from the 1:1 expectation and the original strict clock model shows how non-clock branches change in variance through time [10]. The slope of this regression was used to inform the hyperprior of the variance increase in the IGR model as a median estimate. Using the R scripts of Ronquist *et al.* [10], this slope was found to be 0.022 (figure S1), which gives a median value for the exponential prior of 30.57326 (this value was obtained by ignoring the branch leading to Eutheria, which, as noted above, was a prominent outlier – Fred Ronquist, pers. comm).


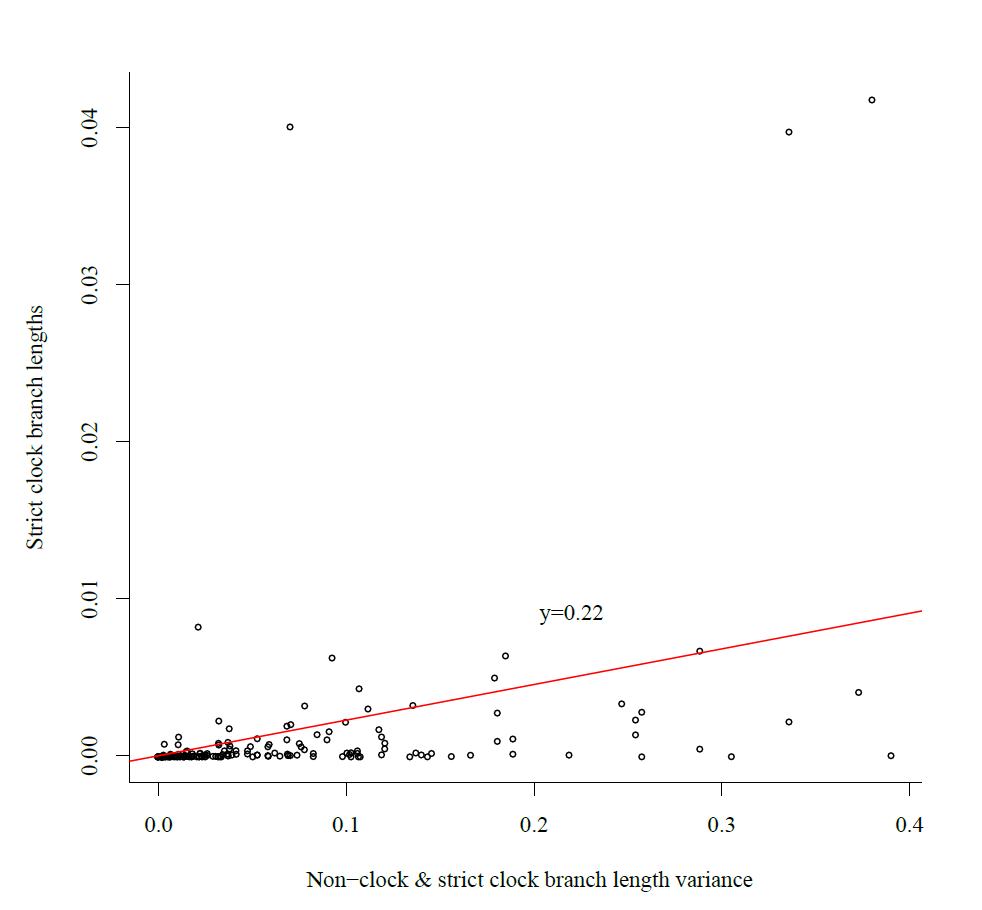


**Figure S1**. A protocol from Ronquist *et al.* [10] was used to produce appropraite priors for the total evidence analyses. A regression of the squared deviation of the non-clock model from the 1:1 expectation and the original strict clock model. The value of this regression is used as the prior for the IGR model in analyses.

Clock substitution rate

To find a suitable prior for the substitution rate of the clock, the fixed topology was run with a strict clock model with different expectations on the prior for the base rate of the phylogeny; these expectations were all exponential distributions with different means: 0.1, 1, and 10 [10]. The chosen value of the median of the posterior was divided by the age of the phylogeny, so a prior for a lognormal distribution of the clock rate could be calculated [10]. The tree height posterior median varied around 0.732 to 0.738 for the different prior distributions; using the posterior from the exponential one distribution divided by the mean age estimation resulted in a clock estimate of 0.00459 substitutions per Ma (this value was used for the clockrate prior in MrBayes) [10].

Autocorrelation

To test the possibility of autocorrelation in rates in the tree, the ratios of ancestor-descendent branch rates were compared to pairs of random branches in the phylogeny [10]. The ratios are the substitution branch length in the ancestor branch divided by the strict clock rate over the substitution rate divided by strict-clock rate in the descendant. Comparing the distributions of the two rate ratios (ancestor-descendants and random pairs of branches) by using the Kolmogorov-Smirnov test indicates that the two distributions are not significantly different from each other (p = 0.4267, D = 0.0937), suggesting there is not a large amount of autocorrelation in the data. However, plotting a histogram of the data shows that the ratios of parent-offspring branches are generally smaller than random branches, which shows there is some autocorrelation in the data [10].

1. **Convergence**

For all models, convergence was tested using MrBayes’ in-built convergence diagnostics [11], specifically, the average standard deviation between runs, the effective sample size (ESS), and the potential scale reduction factor (PSRF). ESS measures the sampling that took place during the MCMC run; a low ESS indicates high auto-correlation between samples in the run, so the posterior was not sampled independently from the distribution during the run. A target figure for ESS was over 200 [10]. The PSRF is used to gauge how much a posterior distribution may decrease with increased runs, and at convergence should approach 1; in these analyses, convergence is judged to have been reached when PSRF reaches 1.02 [10]. The final measure of convergence is the standard deviation in split frequencies that measures the number of times a clade is found in the independent runs, and should be below 0.05, or ideally 0.01 [10]. All analyses were also viewed in Tracer [13] to ensure stationarity had been reached.

1. **Phylogenetic Constraints**

To help the analysis to converge, two constraints were employed to separate the species of the Atlantogenata and remaining species on the phylogeny, as well as to link the two Tubulidentata species [6]. Based on an initial topology, further constraints were also introduced in the Hyracoidea and Rhynchoncyoninae, and others for node dating. A constraint was introduced for Hyracoidea; in the initial analyses, all extant species grouped outside the fossil species, and living species are thought to be closely related to *T. domorictus* [14]. This may be a problem, as in the Hyracoidea fossils greatly outnumber the three extant species, and only one of the extant species has morphological data [15]. A constraint for the Hyracoidea ensured the basal species *Seggeurius amourensis, Microhyrax lavocati,* and *Dimaitherium patnaiki* [8, 14] formed an outgroup to the remaining species. A constraint also was introduced to constrain the monophyly of Rhynchoncyoninae.

1. **Temporal Constraints**

Temporal constraints for nodes were based mainly on Benton *et al.* [16] (Supplementary Table 1). For calibration in the tip-dating analysis fossil occurrence dates were downloaded from the FossilWorks database on the 20^th^ November 2013 [17]. Exceptions to this were for *Arcanotherium* [18], *Dilambdogale* (fixed to 37 Ma) [19], *Omanitherium* [8], *Protenrec* (early Miocene) [19], and *Titanohyrax* *andrewsi* was set to late Miocene/early Oligocene. Species from FossilWorks were verified for veracity (see Supplementary Data, below)

| Node | Minimum | Maximum | Reference |
| --- | --- | --- | --- |
| Crown Theria (root) | 157.3 | 169.9 | [16] |
| Marsupialia | 47.6 | 131.3 | [16] |
| Placentalia | 61.6 | 164.6 | [16] |
| Boreoeutheria | 61.6 | 164.6 | [16] |
| Xenarthra | 47.6 | 164.6 | [16] |
| Atlantogenata | 56 | 164.6 | [16] |
| Afrotheria | 56 | 164.6 | [16] |
| Paenungulata | 55.6 | 71.2 | [20] |
| Macroscelidea | 23 | 29 | [21] |

**Table S1.** The minimum and maximum ages of nodes constrained in the analyses, and the reference for which the age justification was used.

**Supplementary Results**

1. **Non-clock phylogeny**

The non-clock phylogeny (figure S2) conforms to many of the recognised groupings for Mammalia. At the higher-levels of phylogeny all major groups are recognised; there are higher-level splits between the Marsupialia, stem-eutherians *Prokennalestes* and *Montanalestes*, and the Eutheria [19], although these taxa have fallen in other positions [6,19]. Within the Atlantogenata [24] the armadillo (*D. novemcinctus*) is sister to the sloth (*C. didactylus*) and the ant-eater (*T. tetradactyla*), as seen in most phylogenies [20]. The eutherian superorders Laurasiatheria and Euarchontoglires are recovered [20,23,24-25], but the order Eulipotyphla (*E. europeus, C. cristata*) is including in the Euarchontoglires in an anomalous position – possibly due to small taxon sampling.

**Afrotheria**

A large difference between the molecular-only (figure S3) and total-evidence non-clock (figure S2) topological analyses is the effects of morphological cladistics data. These differences are manifested in Afroinsectiphilia not being monophyletic in the total-evidence topology, but it is retained in the molecular-only analysis. In the molecular-only dataset the topology conforms to the recent phylogeny of Kuntner *et al.* (2011) [5]: in this phylogeny there is a monophyletic Afroinsectiphilia and Paenungulata. In the total-evidence topological analyses, the Afrosoricida is still monophyletic, but there is a branching pattern in which the following clades diverge: the Macroscelidea, a clade of *Herodotius* and *Chambius* (the Herodontinae), Tubulidentata, and finally the monophyletic Paenungulata [6].

In the molecular-only topology, the phylogenetic grouping of the afrotherian clades is in accordance with most recent studies, with the major split shown between Paenungulata (Proboscidea, Sirenia, Hyracoidea) and Afroinsectiphilia (Macroscelidea, Chrysochloridae, Tenrecidae, Tubulidentata) [5,20,22,23] (Supplementary Figure S3).

The Afroinsectiphilia has Tubulidentata as the sister-group to the rest of the clade in the molecular phylogeny. This differs in the total-evidence analysis with the extant aardvark (*O. afer*) and the extinct *M. africanus* forming a sister clade to the Paenungulata, in a clade called the Pseudoungulata [6]. The remaining orders within the Afroinsectiphilia support the well-known relationship of a sister grouping between the Afrosoricida and Macroscelidea [5,6,23], a clade sometimes known as the Afroinsectivora [6].

**Macroscelidea**

The genus *Elephantulus* has been shown to be non-monophyletic, as *E. rozeti* is most closely related to *Petrodomus tetradactylus*, and together they form a clade with *Macroscelides proboscideus* [21,26,27]; these three species and the remaining *Elephantulus* species form a clade, sometimes called the Macroscelidinae [21,27]. The Macroscelidinae then form a sister group to the Rhynchocyoninae [21,27] to make up the rest of the extant Macroscelidea. However, there are some differences in the phylogeny here; three *Elephantulus* species (*E. fuscus, E. revioli, E. fuscipes*) are found in the Rhynchoncyoninae; the three *Elephantulus* species are typically species that group outside the main Macroscelidinae clade [21,27], but the split between Rhynchoncyoninae and Macroscelidinae is supported by both molecular and morphological data [21,27]. One reason for this relationship may be the apparently high rate of evolution in *E. fuscus* and *E. revioli*. The fossil taxa *Metoldobotes* is an outgroup to the Rhynchyoncyoninae [6]. Whilst *Chambius* is sometimes place as basal member of the Macroscelidea [6], here it has also be placed as a pseudoungulate alongside *Herotodius* in the Herodontinae [6,19].

**Afrosoricida**

In the Afrosoricida, many of the expected relationships at the species level are found. Tenrecs and Golden Moles were long classified as ‘Insectivora’ until molecular phylogenetics confirmed that these orders belong in Afrotheria [28]. Golden Moles (Chrysochloridae) are known to be morphologically distinct from other mammals, and even other Afrotheres [4]. Comparisons with other studies can be difficult as many studies have not had good sampling for the Golden moles [5,6], but others have [4]. In the phylogeny here, the grouping of the genera (*Amblysomus, Neamblysomus,* and *Carpitalpa*) into the clade Amblysominae is supported [4, 29]. However the remaining species within the order form no obvious groups, and do not form a monphyletic group to a clade that is known as Chrysochlorinae [4,29]. Instead, Chrysochlorinae is paraphyletic, as shown previously [4]. The phylogeny here is most similar to Asher [4], as the origin of the clade is placed between *Calcochloris*, *Cryptochloris*, and *Chrysochcloris*, and the rest of the Golden Moles; a slight difference is that the origin of the Golden Moles in Asher [4] was placed with those three genera and *Eremitalpa*. The groupings of the species in the genera *Chrysospalax* and *Chlorotalpa* are also seen, and there is no grouping between *Calcochloris leucorhina* and *Calcochloris obtusirostris*, giving further weight to the idea that *Calcochloris leucorhina* should be known as the genus *Huetia* [4]; additionally, as *Carpitalpa arendsi* groups with the *Neamblyosomus* genus, this too gives extra weight to it being classified as a separate genus [4].

Of the extant Tenrecidae, our phylogeny agrees with most recent phylogenies: the African mainland species (*Potamogale velox* and *Micropotamogale lamottei*) form the outgroup to the remaining species, the Madagascan ‘Malagasy’ tenrecs [5,30,31]. Of the Malagasy tenrecs, we also find the Tenrecinae, Geogalinae, and Oryzoctinae subclades [5,31], although these subclades are not always identified [30]. Placement of fossil taxa in the phylogeny does not agree with the findings of Asher & Hofreiter [30], although they included two fossil species not included here (*Erythrozootes* and *Parageogale*), their phylogeny positioned *Protenrec* as a nested within the Malagasy tenrecs as sister to *Geogale.* Here *Protenrec* is found in a clade with the extant Mainland tenrecs (*P. velox* and *M. lamottei*), which agrees with Seiffert [6]. This is potential a more parsimonious suggestion as the Eocene *Protenrec* from Kenya is more basally situated, and not sister to a species nested in a Madagascan monophyletic clade [30]. The basal tenrec species are included in a clade with *Protenrec, Dilambdogale*, and *Widanelfarasia* and the two extant mainland species. The inclusion of *Dilambdogale* and *Wildanelfarasia* species from the Eocene and Oligocene here confirms there position as basal Tenrecoids, rather than basal Afrosorocida [19]; the difference in the location of *Dilambdogale* and *Wildanefarasia* is likely the result of greater taxon sampling, compared to previous analyses [8, 19].

**Paenungulata**

The relationship between the orders Sirenia, Proboscidea, and Hyracoidea are now firmly believed to be the only extant orders in the Paenungulata clade [5, 7,19,20]. However, the relationship between those three orders has not been clarified; a consensus may be that the Sirenia and Proboscidea form a sister-group relationship [6,22,32], but the other two potential groupings have also been found. For example, support has been given to a Proboscidea + Hyracoidea relationship [20], and the Hyracoidea + Sirenia grouping [22, 33]. The Sirenia + Hyracoidean grouping is one which finds support here through use of the molecular-only datasets, but the Sirenia + Proboscidea is found in the total-evidence topology [6,22,32].

**Hyracoidea**

Hyracoidea in our phylogeny out represented by a large number of fossil species, and just three extant species: the western tree hyrax *Dendrohyrax dorsalis,* yellow-spotted rock hyrax (*Heterohyrax brucei*)*,* and the rock hyrax (*Procavia capensis*). In the original unconstrained analyses, a rather surprising relationship emerges in which *H. brucei* and *P. capensis* form outgroups to a clade containing *D. dorsalis* and the remaining fossil groups. Previous analyses have placed *P. capensis* as sister to the genus *Thyrohyrax* but here *P. capensis* groups with *H. brucei* outside the main hyrax clade, but *Thyrohyrax* is still shown to be paraphyletic [6,8]. The fossil clades *Titanohyrax, Antilohyrax,* and *Afrohyrax* are still recovered [8]. *Dimatherium, Microhyrax,* and *Segguerius* are still found to be a basal clade [8], and a derived clade of *Megalohyrax, Bunohyrax, Geniohyrax,* and *Pachyhyrax* is still shown in a derived postion [6,8]. Many of these relationships do not fit stratigraphically, as the Eocene *Seggeurius* and *Microhyrax* are placed nested with the Hyracoidea [6]. In the subsequent analyses, a constraint was introduced (see above).

**Sirenia**

The extinct species *Prorastomus* and *Protosiren* are shown as basal members of the clade, whilst the third extinct Sirenia included in the analyses, *Hydrodamalis gigas*, is placed as sister to the dugong [5], whilst the split between *Trichechus* and *Dugong* is maintained [34]. Whilst this includes some basal members of Sirenia, it is not exhaustive, as species such as *Pezosiren* have not been included.

**Proboscidea**

The phylogeny of Proboscidea largely agrees with the findings of Seiffert *et al.* (2012) [8]. Unlike Seiffert *et al.* [8] the Oligocene *Omanitherium* is placed as sister taxa to *Arcanotherium* not *Barytherium*. A sister-group exist between early Proboscidean Plesielephantiformes and a clade containing all extant species, the Elephantiiformes; within Plesielephantiformes their exist a split between a clade containing the Bartheriidae and Moerithidae (*Barytherium, Arcanotherium, Omaniherium*) and Numidotheriidae (*Numidotherium, Daoutherium, Phosphatherium*) which is a pattern that has not been seen in previous uses of this dataset [6,8,19]. In the Elephantiiformes, a surprising result groups *Phiomia* and *Elephas maximus*, which may be due to high rates of evolution on these branches (and is not seen in the dated analyses – see below). The topology of the remaining genera conforms to expectations as in the Elephantidae the extant *Loxodonta* species branch, followed by a sister-group relationship of *Elephas*, which includes the Indian elephant (*E. maximus*) and *Mammuthus* [5,35].


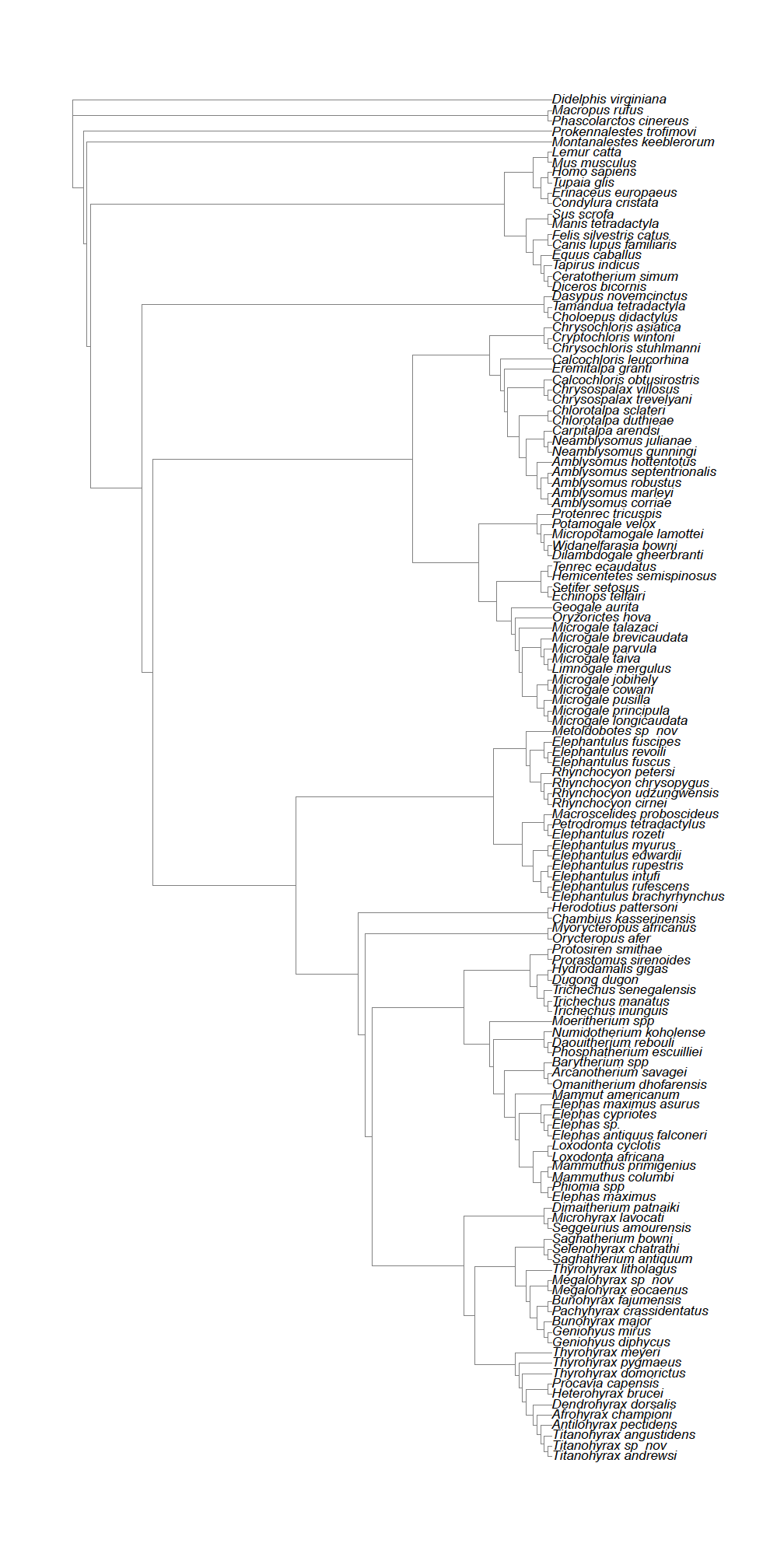


**Figure S2**. Total-evidence non-clock topology for the total evidence dataset using only constraints on the Atlantogenata, Tubulidentata, Hyracoidea, and Rhynchoncyoninae.


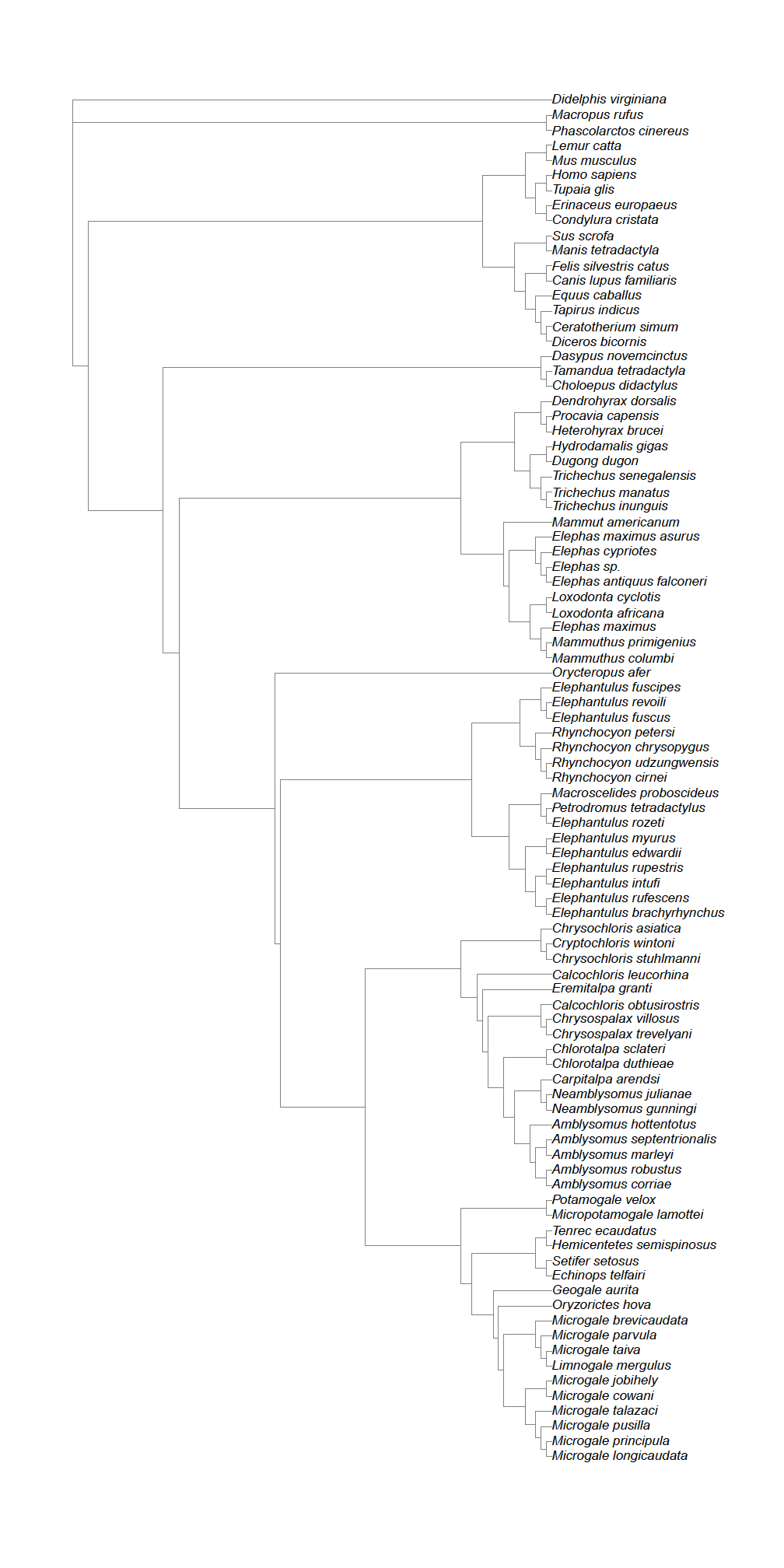


**Figure S3**. Molecular-only non-clock topology for the total evidence dataset using only constraints on the Atlantogenata, Tubulidentata, Hyracoidea, and Rhynchoncyoninae.

1. **Total-evidence phylogeny**

The total evidence phylogeny (figure 1 and S4) is similar to the non-clock phylogeny (figure S2). The composition of all the major clades is identical and the inter-ordinal differences very similar. The largest differences are seen in the position of the afrosoricid fossils *Dilambdogale gheerbranti* and *Wildanefarasia bowni*, which move from the derived position of sister to *M. lamottei* to the stem of the Chrysochloridae. Additionally, the species, the Heterodontidae (*Chambius* and *Heterodontius*) form a sister group to the Tubulidentata as they do in Seiffert *et al.* (2012).


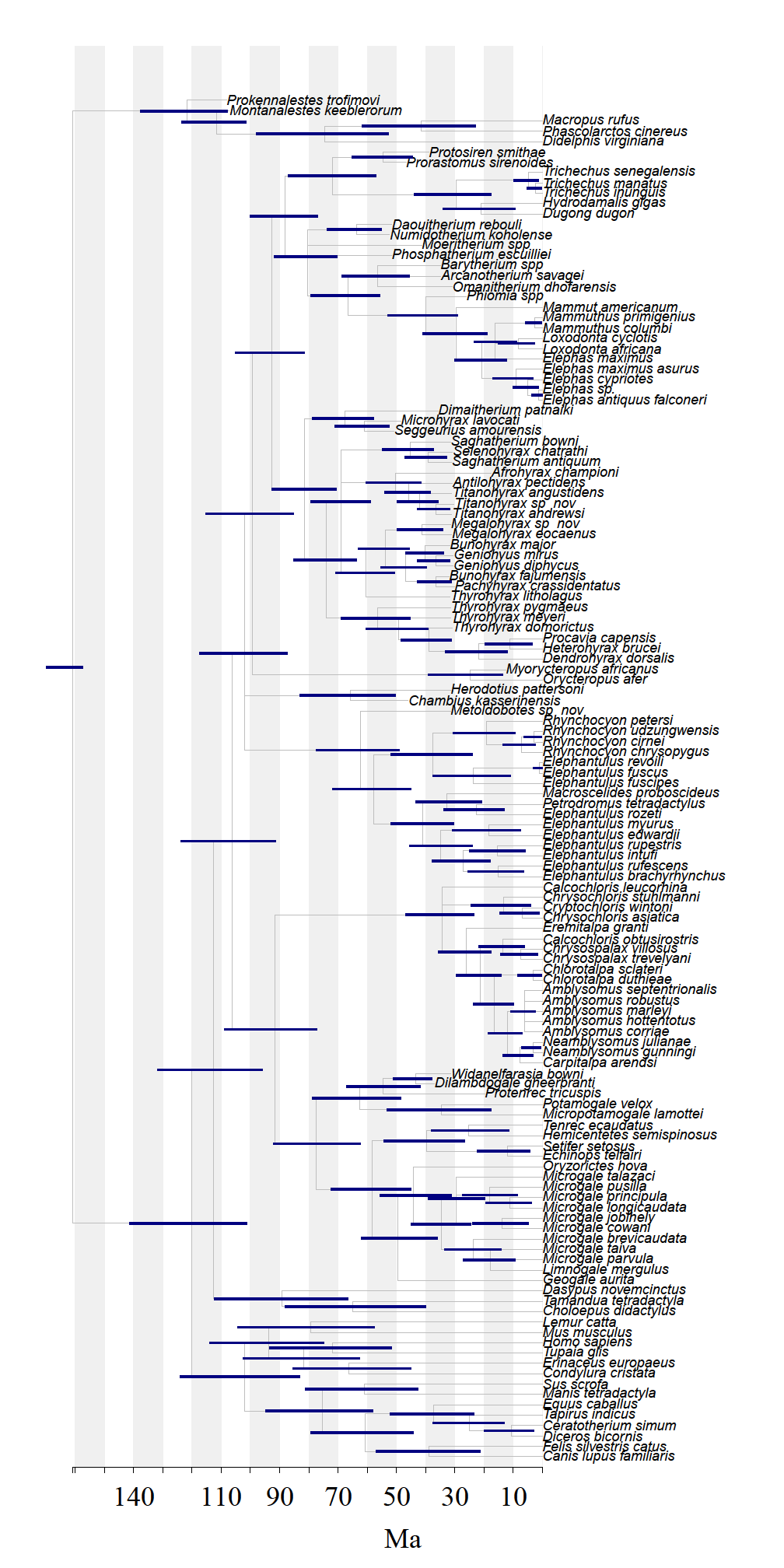


**Figure S4**. Total-evidence phylogeny constrained with off-set exponential node calibrations and uniform-based tip priors. The blue lines represent the 95% posterior densities.

**9. Node-dating phylogeny**

The differences in the molecular only (figure S4) and molecular plus morphological node dated phylogeny (figure S5) reflect the differences in the non-clock analyses; when morphology is included, the Afroinsectiphilia is no longer monophyletic as the Macroscelidea forms an outgroup to the Pseudopaenungulata (figure S5). Furthermore, the position of the Tubulidentata has changed with *Orycteropus afer* forming a sister clade to the Paenungulata.


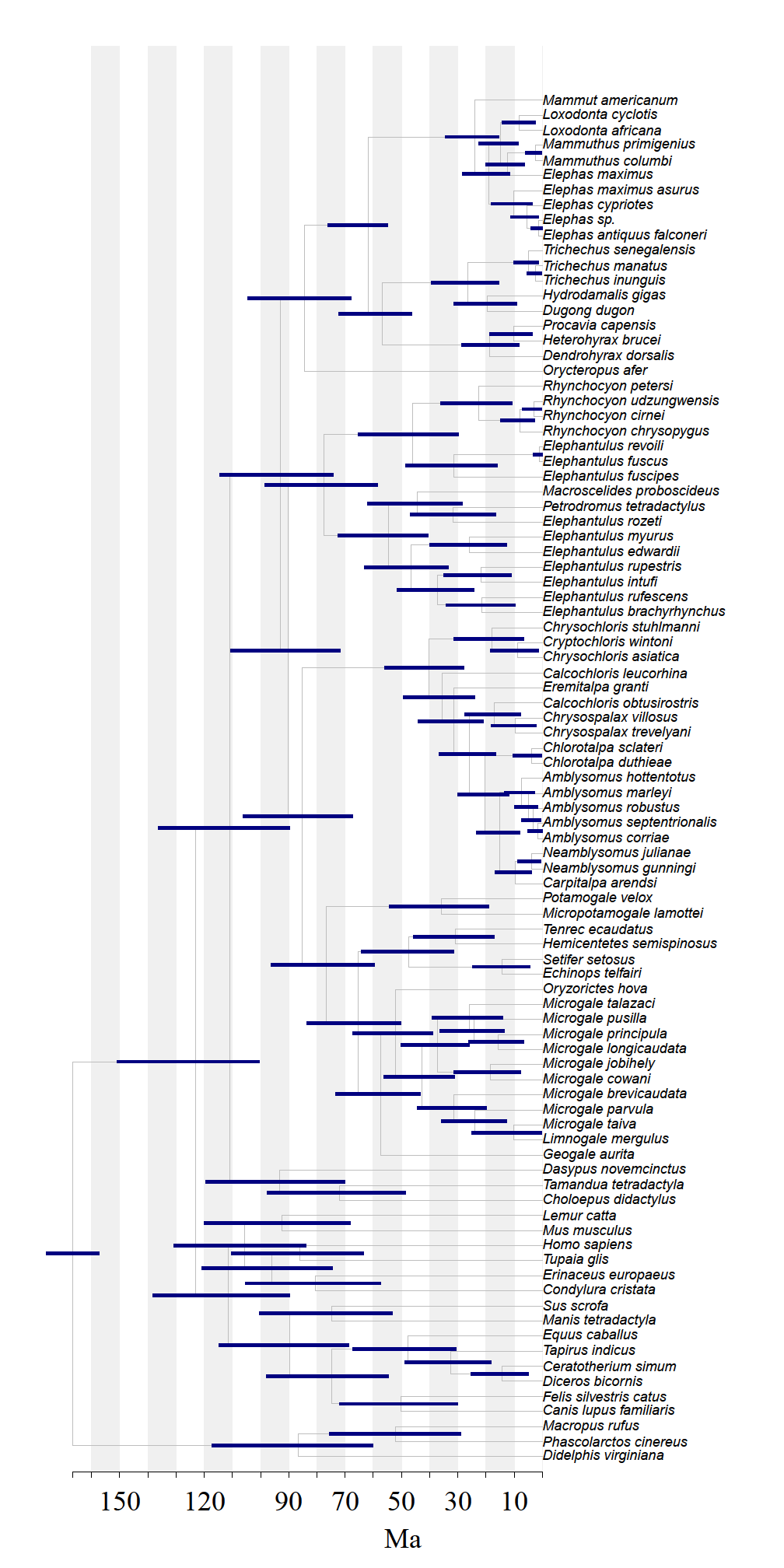


**Figure S5.** Node-dated constrained phylogeny based on molecular data only with off-set exponential node calibrations. The blue lines represent the 95% posterior densities.


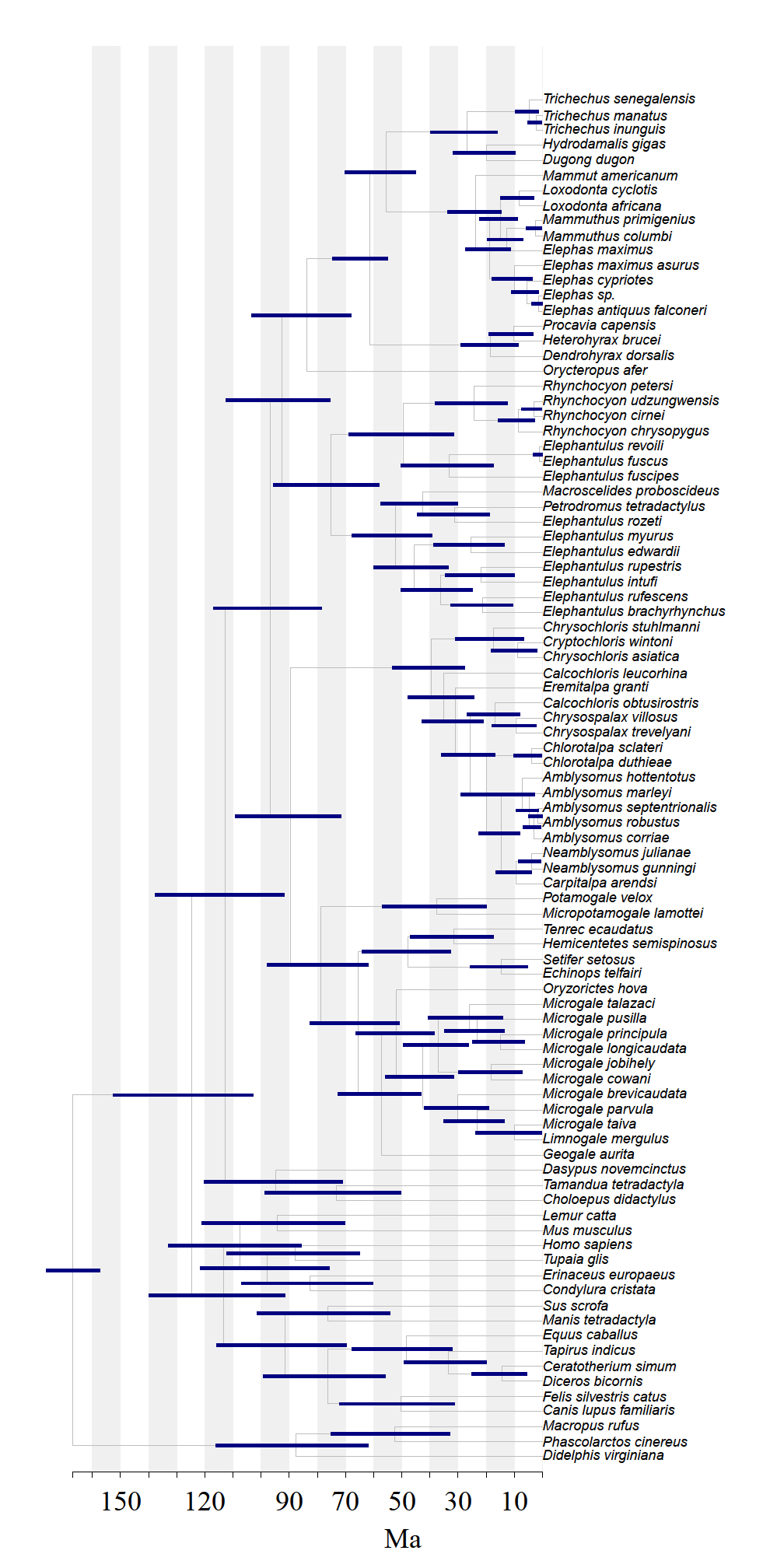


**Figure S6.** Node-dated constrained phylogeny based on molecular and morphological data with off-set exponential node calibrations. The blue lines represent the 95% posterior densities.

**10. Comparison of total-evidence and molecular ages**

**Figure S6.** Estimated ages from node dating are generally younger than total-evidence ages. All ages from the total-evidence analysis for afrotherian clades are older than the constrained molecular node-dated phylogeny (left) and the combined molecular and morphology node-dating dataset (right). The red line is the best-fitting relationship and the grey lines around the points indicate the 95% posterior intervals of the age estimates.


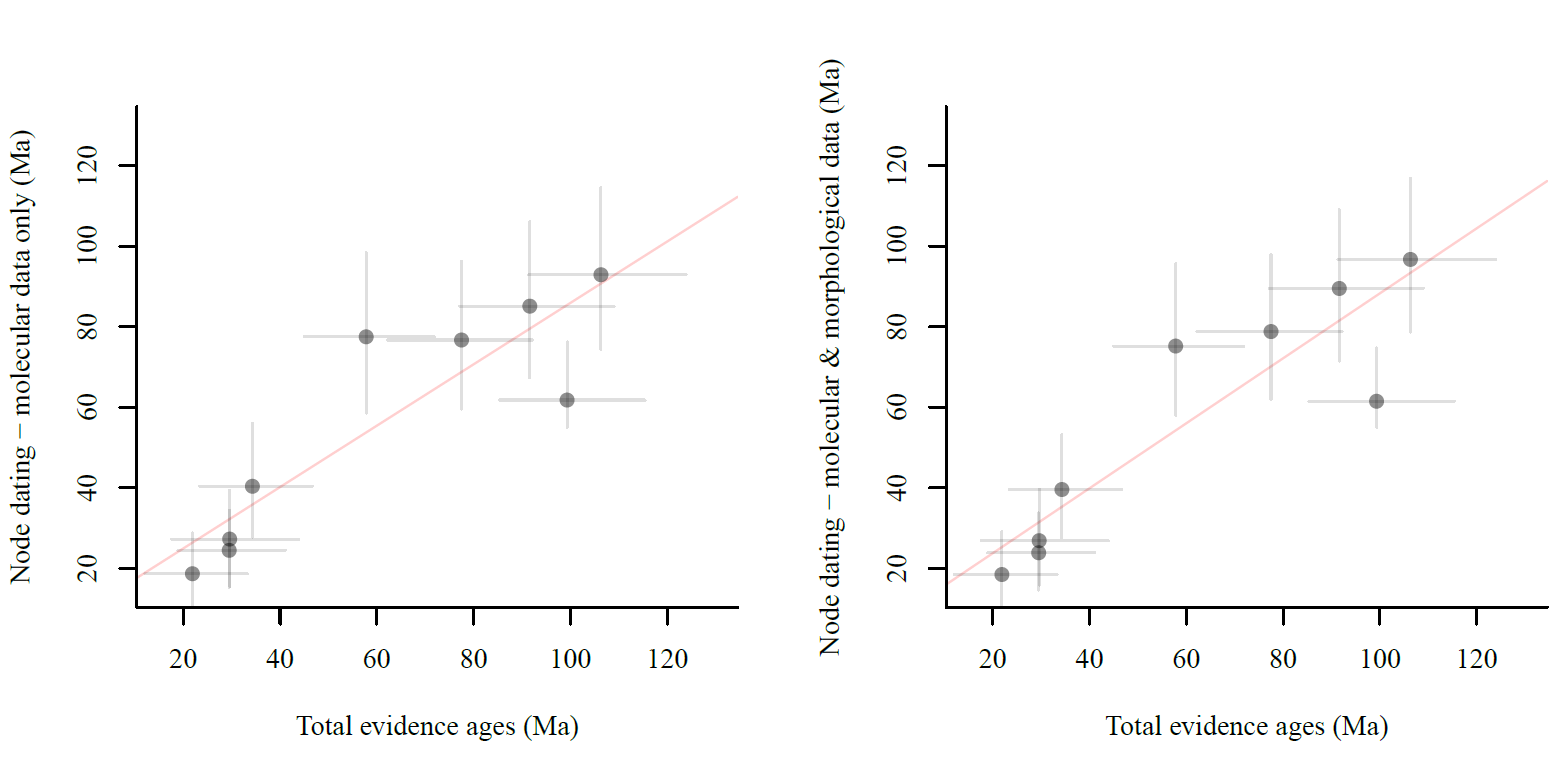


| Outgroup Mass (kg) | Est. Root Mass (kg) | Afrotheria Ancestral Mass (kg) | BM Est. Root Mass (kg) | BM Afrotheria Ancestral Mass (kg) | ΔPBIC (BM – Stable) |
| --- | --- | --- | --- | --- | --- |
| 0.1 | **0.1** (0.10-0.10) | **0.10** (0.03-0.31) | **0.10** (0.09-0.11) | **0.48** (0.15-1.58) | 32.5055 |
| 0.5 | **0.5** (0.48-0.52) | **0.27** (0.06-1.02) | **0.5** (0.48-0.53) | **0.93** (0.28-2.99) | 22.1525 |
| 1 | **1** (0.97-1.03) | **0.43** (0.07-1.89) | **1** (0.95-1.05) | **1.24** (0.38-4) | 18.042 |
| 5 | **5** (4.83-5.16) | **1.35** (0.05-9.35) | **5** (4.76-5.25) | **2.45** (0.75-8.03) | 15.046 |
| 10 | **10** (9.67-10.31) | **2.03** (0.04-18.42) | **9.99** (9.51-10.5) | **3.24** (0.99-10.71) | 16.756 |
| 20 | **20** (19.37-20.61) | **2.02** (0.04-36.28) | **19.98** (19.01-21) | **4.35** (1.31-14.54) | 24.146 |

**11. Effects of prior information on ancestral state estimation**

**Supplementary Table S2.** The use of outgroup priors in the StableTraits model produces ancestral Afrotheria masses that are comparable to the total-evidence phylogeny, and the ancestral mass for Afrotheria is considerably smaller than the outgroup mass.

| Outgroup Mass (kg) | Est. Root Mass (kg) | Afrotheria Ancestral Mass (kg) | BM Est. Root Mass (kg) | BM Afrotheria Ancestral Mass (kg) | ΔPBIC (BM – Stable) |
| --- | --- | --- | --- | --- | --- |
| Original Stem (5 Myr) | | | | | |
| 0.1 | **0.1** (0.10-0.10) | **0.1** (0.04-0.32) | **0.1** (0.10-0.10) | **0.38** (0.11-1.33) | 62.8855 |
| 0.5 | **0.5** (0.49-0.51) | **0.38** (0.28-1.25) | **0.5** (0.48-0.52) | **0.88** (0.26-2.89) | 45.3835 |
| 1 | **1** (0.98-1.02) | **0.69** (0.09-2.43) | **1** (0.96-1.05) | **1.24** (0.37-4.09) | 38.3115 |
| 5 | **5** (4.89-5.11) | **3.42** (0.08-14.46) | **5** (4.78-5.23) | **2.85** (0.85-9.56) | 45.3765 |
| 10 | **10** (9.8-10.19) | **7.55** (0.09-31.26) | **9.99** (9.54-10.46) | **4.03** (1.19-13.8) | 47.0085 |
| 20 | **20** (19.61-20.4) | **15.75** (0.09-60.7) | **19.98** (19.07-20.92) | **5.81** (1.64-19.96) | 42.515 |
| Short Stem (1 Myr) | | | | | |
| 0.1 | **0.1** (0.10-0.10) | **0.1** (0.08-0.13) | **0.1** (0.10-0.11) | **0.12** (0.07-0.19) | 69.135 |
| 0.5 | **0.5** (0.49-0.51) | **0.49** (0.33-0.66) | **0.5** (0.48-0.52) | **0.54** (0.35-0.83) | 42.4745 |
| 1 | **1** (0.98-1.02) | **0.98** (0.58-1.33) | **1** (0.96-1.05) | **1.03** (0.66-1.59) | 37.3965 |
| 5 | **5** (4.89-5.11) | **4.94** (2.73-6.7) | **5** (4.78-5.23) | **4.65** (2.98-7.22) | 37.2515 |
| 10 | **10** (9.79-10.2) | **9.91** (5.37-13.46) | **9.99** (9.53-10.47) | **8.88** (1.19-13.8) | 38.401 |
| 20 | **20** (19.62-20.39) | **19.91** (12.52-26.99 | **19.96** (19.03-20.93) | **16.98** (10.66-27.0) | 47.3435 |

**Supplementary Table S3.** The use of outgroup priors in the StableTraits model with only extant taxa produces ancestral Afrotheria masses that are smaller than the prior (top). When a short stem (1 Myr) to the outgroup is used, the ancestral Afrotheria mass estimate is much closer to the prior.

**Supplementary Figure S7.** The use of outgroup priors in the StableTraits model has minimal impact on the estimate rates on the phylogeny: the red line indicates a large increase in rate leading to the Paenungulata and Tubulidentata in all analyses.


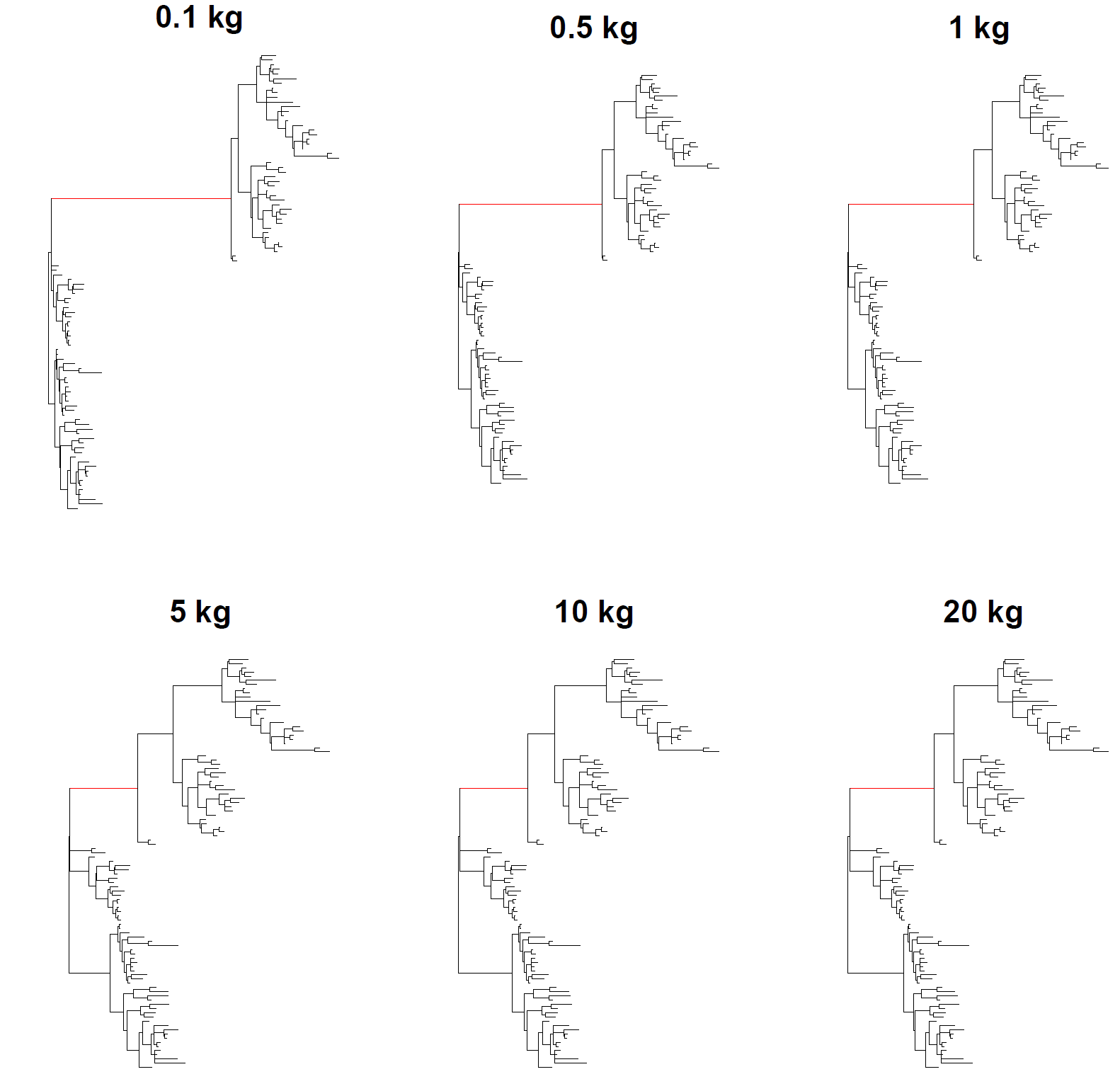


**12. Supplementary References**

1. Wilson DE, DA Reeder, eds. 2005 Mammal species of the world. Johns Hopkins Univ. Press, Baltimore, MD

2. Goodman SM, Raxworthy CJ, Maminirina CP, Olson LE. 2006 A new species of shrew tenrec (Microgale jobihely) from northern Madagascar. *J. Zool.* **270**, 384-398.

3. Rovero F, Rathbun GB, Perkin A, Jones T, Ribble DO, Leonard C, Mwakisoma RR, Doggart N. 2008. A new species of giant sengi or elephant‐shrew (genus Rhynchocyon) highlights the exceptional biodiversity of the Udzungwa Mountains of Tanzania. *J. Zool.* **274**, 126-133.

4. Asher RJ, Maree S, Bronner G, Bennett NC, Bloomer P, Czechowski P, Meyer M, Hofreiter, M. 2010 A phylogenetic estimate for golden moles (Mammalia, Afrotheria, Chrysochloridae). *BMC Evol. Biol.* **10**, 69. (doi:10.1186/1471-2148-10-69)

5. Kuntner M, May‐Collado LJ, Agnarsson I. 2011 Phylogeny and conservation priorities of afrotherian mammals (Afrotheria, Mammalia). *Zool. Scripta*. **40**, 1-15.

6. Seiffert ER. 2007 A new estimate of afrotherian phylogeny based on simultaneous analysis of genomic, morphological, and fossil evidence. *BMC Evol. Biol.* **7**, 224. (doi: 10.1186/1471-2148-7-224)

7. Tabuce R, Asher RJ, Lehmann T. 2008 Afrotherian mammals: a review of current data. *Mammalia* **72**, 2-14.

8. Seiffert ER, Nasir S, Al-Harthy A, Groenke JR, Kraatz BP, Stevens NJ, Al-Sayigh AR. 2012 Diversity in the later Paleogene proboscidean radiation: a small barytheriid from the Oligocene of Dhofar Governorate, Sultanate of Oman. *Naturwissenschaften* **99**,133-141.

9. Lewis PO. 2001 A likelihood approach to estimating phylogeny from discrete morphological character data. *Syst*. *Biol*. **50**, 913-925.

10. Ronquist F, Klopfstein S, Vilhelmsen L, Schulmeister S, Murray, DL, Rasnitsyn, AP 2012 A total-evidence approach to dating with fossils, applied to the early radiation of the Hymenoptera. *Syst. Biol*. **61**, 973-999.

11. Ronquist F, Teslenko M, van der Mark P, Ayres, DL, Darling A, Höhna S, Larget B, Liu L, Suchard MA, Huelsenbeck JP. 2012 MrBayes 3.2: efficient Bayesian phylogenetic inference and model choice across a large model space. *Syst. Biol.* **61**, 539-542.

12. Lepage T, Bryant D, Philippe H, Lartillot N. 2007 A general comparison of relaxed molecular clock models. *Mol*. *Biol*. *Evol*. **24**, 2669-2680.

13. Rambaut A, Suchard MA, Xie D, Drummond AJ. 2014 Tracer v1.6, Available from <http://beast.bio.ed.ac.uk/Tracer>

14. Barrow EC, Seiffert ER, Simons EL. 2012 Cranial morphology of Thyrohyrax domorictus (Mammalia, Hyracoidea) from the early Oligocene of Egypt. *J. Vert. Paleontol*. **32**, 166-179.

15. Wiens JJ, Kuczynski, CA, Townsend T, Reeder TW, Mulcahy DG, Sites JW. 2010 Combining phylogenomics and fossils in higher-level squamate reptile phylogeny: molecular data change the placement of fossil taxa. *Syst. Biol.* **59**, 674-688.

16. Benton MJ, Donoghue PCJ, Asher RJ, Friedman M, Near TJ, Vinther, J. 2015 Constraints on the timescale of animal evolutionary history. *Palaeontol. Electron.* **18.1.1FC**, 1-106; palaeo-electronica.org/content/fc-1

17. Alroy J, Uhen MD, Behrensmeyer AK, Turner A. 2013 Taxonomic occurrences of Afrotheria recorded in Fossilworks, the Evolution of Terrestrial Ecosystems database, and the Paleobiology Database. Fossilworks. [http://fossilworks.org](http://fossilworks.org/).

18. Delmer C. 2009 Reassessment of the generic attribution of Numidotherium savagei and the homologies of lower incisors in proboscideans. *Acta* *Palaeontol*. *Pol*. **54**, 561-580.

19. Seiffert ER. 2010 The oldest and youngest records of afrosoricid placentals from the Fayum Depression of northern Egypt. *Acta* *Palaeontol*. *Pol*. **55**, 599-616.

20. Meredith RW, *et al.* 2011 Impacts of the Cretaceous terrestrial revolution and KPg extinction on mammal diversification. Science **334**, 521–524.

21. Smit HA, Jansen van Vuuren B, O'Brien PCM, Ferguson‐Smith M, Yang F, Robinson TJ. 2011 Phylogenetic relationships of elephant‐shrews (Afrotheria, Macroscelididae). *J. Zool.* **284**, 133-143.

22. Murphy WJ, *et al.* 2001. Resolution of the early placental mammal radiation using Bayesian phylogenetics. Science **294**, 2348–2351.

23. Bininda-Emonds ORP, Cardillo M, Jones KE, MacPhee RDE, Beck RMD, Grenyer R, Price SA, Vos RA, Gittleman JL, Purvis A. 2007 The delayed rise of present-day mammals. *Nature* **446**, 507-512.

24. dos Reis M, Inoue J, Hasegawa M, Asher RJ, Donoghue PCJ, Yang Z. 2012 Phylogenomic datasets provide both precision and accuracy in estimating the timescale of placental mammal phylogeny. Proc. R. Soc. B: Biol. Sci. **279**, 3491–3500.

25. Zhou X, Xu S, Xu J, Chen B, Zhou K, Yang G. 2012 Phylogenomic analysis resolves the interordinal relationships and rapid diversification of the Laurasiatherian mammals. *Syst. Biol.* **61**, 50-164.

26. Douady CJ, Catzeflis F, Raman J, Springer MS, Stanhope MJ 2003 The Sahara as a vicariant agent, and the role of Miocene climatic events, in the diversification of the mammalian order Macroscelidea (elephant shrews). *Proc. Natl. Acad. Sci. U S A.* **100**, 8325-8330.

27. Benoit J, Crumpton N, Merigeaud S, Tabuce R. 2014. Petrosal and Bony Labyrinth Morphology Supports Paraphyly of Elephantulus Within Macroscelididae (Mammalia, Afrotheria). *J. Mamm. Evol.* **21**, 173-193.

28. Symonds MR. 2005 Phylogeny and life histories of the ‘Insectivora’: controversies and consequences. *Biol. Rev.* **80**, 93-128.

29. Bronner GN, Jenkins PD. 2005 Order Afrosoricida. Mammal species of the world, 71-77.

30. Asher RJ, Hofreiter M. 2006 Tenrec phylogeny and the noninvasive extraction of nuclear DNA. *Syst. Biol*. **55**, 181-194.

31. Poux C, Madsen O, Glos J, De Jong WW, Vences M. 2008. Molecular phylogeny and divergence times of Malagasy tenrecs: influence of data partitioning and taxon sampling on dating analyses. *BMC Evol. Biol.* **8**, 102. (doi: doi:10.1186/1471-2148-8-102)

32. Stanhope MJ, Madsen O, Waddell VG, Cleven GC, de Jong, WW. 1998 Highly congruent molecular support for a diverse superordinal clade of endemic African mammals. *Mol. Phylogenet. Evo*. **9**, 501-508.

33. Nishihara, H., Hasegawa, M., and Okada, N. 2006. Pegasoferae, an unexpected mammalian clade revealed by tracking ancient retroposon insertions. Proc. Natl. Acad. Sci. USA 103: 9929-9934.

34. Ozawa T, Hayashi S, Mikhelson VM. 1997 Phylogenetic position of mammoth and Steller's sea cow within Tethytheria demonstrated by mitochondrial DNA sequences. *J. Mol. Evol*. **44**, 406-413.

35. Shoshani J, Tassy P. 2005 Advances in proboscidean taxonomy & classification, anatomy & physiology, and ecology & behavior. *Quatern. Int.* **126**, 5-20.

**Fossil Data**

**13. Measurements and sources**

The predominate equation was the regression equation for mammals from Bloch *et al.* 1998 [1] for lower molar area (1.628*Ln(m1area)+1.726). Although some Afrotheres may have highly derived dentition, such as Proboscidea, these equations were only used on basal forms. Before analyses, all data were natural log-transformed.

| Species | Mass (g) | Source |
| --- | --- | --- |
| *Geogale aurita* | 6.69 | [2] |
| *Limnogale mergulus* | 92 | [3] |
| *Microgale brevicaudata* | 8.9 | [4] |
| *Microgale cowani* | 13.8 | [4] |
| *Microgale jobihely* | 10.0 | [4] |
| *Microgale longicaudata* | 8.08 | [2] |
| *Microgale parvula* | 3.2 | [4] |
| *Microgale principula* | 10.2 | [2] |
| *Microgale pusilla* | 3.5 | [4] |
| *Microgale taiva* | 11.8 | [4] |
| *Microgale talazaci* | 48.2 | [3] |
| *Oryzorictes hova* | 44.2 | [3] (from Oryzorictes talpoides) |
| *Micropotamogale lamottei* | 78 | [5] (page 218) |
| *Potamogale velox* | 656 | [5] (page 222) |
| *Echinops telfairi* | 87.5 | [3] |
| *Hemicentetes semispinosus* | 116 | [3] |
| *Setifer setosus* | 273 | [3] |
| *Tenrec ecaudatus* | 852 | [3] |
| *Protenrec tricuspis* | 3.14 | Skull length [6] using equation [7] |
| *Carpitalpa arendsi* | 51.5 | [5] (page 238) |
| *Chlorotalpa duthieae* | 29.5 | [5] (vol. 1 page 240) |
| *Chlorotalpa sclateri* | 34.45 | [5] (vol 1 page 241) |
| *Chrysochloris asiatica* | 49 | [3] |
| *Chrysochloris stuhlmanni* | 42 | [5] (vol 1 page 245) |
| *Chrysospalax trevelyani* | 460 | [5] (vol 1 page 248) |
| *Chrysospalax villosus* | 117.85 | [5] (vol 1 page 250) |
| *Cryptochloris wintoni* | NA | NA |
| *Eremitalpa granti* | 23 | [5] (vol 1 page 254) |
| *Amblysomus corriae* | 51.5 | [5] (p 227) |
| *Amblysomus hottentotus* | 53.5 | [5] (p 229) |
| *Amblysomus marleyi* | NA | NA |
| *Amblysomus robustus* | 76 | [5] (p232) |
| *Amblysomus septentrionalis* | 69 | [5] (p233) |
| *Calcochloris leucorhina* | NA | NA |
| *Calcochloris obtusirostris* | 26.3 | [5] (p236) |
| *Neamblysomus gunningi* | 59.6 | [5] (p256) |
| *Neamblysomus julianae* | 28 | [5] (p257) |
| *Dilambdogale gheerbranti* | 10.8 | Molar area [8] using equation of [1] |
| *Widanelfarasia bowni* | NA | NA |
| *Elephantulus brachyrhynchus* | 45.3 | Benoit *et al.* 2014 |
| *Elephantulus edwardii* | 49.25 | [5] (p266) |
| *Elephantulus fuscipes* | 57 | [3] |
| *Elephantulus fuscus* | 45.5 | [5] (p268) |
| *Elephantulus intufi* | 45.8 | [5] (p269) |
| *Elephantulus myurus* | 45.1 | [3] |
| *Elephantulus revoili* | NA | NA |
| *Elephantulus rozeti* | 45.3 | [5] (page 273) |
| *Elephantulus rufescens* | 57.3 | [5] (page 275) |
| *Elephantulus rupestris* | 61.5 | [9] |
| *Macroscelides proboscideus* | 38.2 | [5] (page 278) |
| *Petrodromus tetradactylus* | 198.3 | [5] (page 281) |
| *Rhynchocyon chrysopygus* | 534.8 | [5] (page 284) |
| *Rhynchocyon cirnei* | 352 | [5] (page 286) |
| *Rhynchocyon petersi* | 471 | [3] |
| *Rhynchocyon udzungwensis* | 710 | [3] |
| *Metoldobotes sp nov* | 375.4 | Skull size [10] using equation of Luo et al [7] |
| *Chambius kasserinensis* | 71.2 | Lower molar 1 area [11] equation [1]. |
| *Herodotius pattersoni* | 244.4 | Lower molar 1 area (Tabuce *et al.* 2012) equation [1]. |
| *Orycteropus afer* | 52350 | [5] (page 295) |
| *Myorycteropus africanus* | 28792.50 | [12] (About half size of extant species) |
| *Dendrohyrax dorsalis* | 3175 | [5] (page 157) |
| *Heterohyrax brucei* | 2402 | [5] (page 165) |
| *Procavia capensis* | 3020 | [5] (page 171) |
| *Thyrohyrax domorictus* | 10900 | [13] |
| *Thyrohyrax litholagus* | 22200 | [13] |
| *Thyrohyrax meyeri* | 6500 | [13] |
| *Thyrohyrax pygmaeus* | NA | NA |
| *Titanohyrax andrewsi* | NA | NA |
| *Titanohyrax angustidens* | 262700 | [13] |
| *Titanohyrax sp nov* | NA |  |
| *Saghatherium antiquum* | 16826.7 | Length of m2 [14] using equation of Janis 1990 [16] (perissodactyle-hyracoid) |
| *Saghatherium bowni* | 9200 | [13] |
| *Seggeurius amourensis* | 2932 | [9] |
| *Selenohyrax chatrathi* | 45900 | [13] |
| *Pachyhyrax crassidentatus* | 134200 | [13] |
| *Microhyrax lavocati* | 3400 | [13] |
| *Megalohyrax eocaenus* | 155400 | [13] |
| *Megalohyrax sp nov* | NA | NA |
| *Geniohyus diphycus* | NA | NA |
| *Geniohyus mirus* | 83109.6 | Length of m2 [15] equation of Janis [16] (perissodactyl-hyracoid) |
| *Dimaitherium patnaiki* | 14373.7 | Length of m2 [17] equation of Janis [16] (perissodactyl-hyracoid) |
| *Bunohyrax fajumensis* | 82300 | [13] |
| *Bunohyrax major* | 232200 | [13] |
| *Afrohyrax championi* | 82300 | [13] |
| *Antilohyrax pectidens* | 55884.4097 | Lower m2 length [18] equation of Janis [16] (perissodactyl-hyracoid) |
| *Elephas antiquus falconeri* | 100000 | [19] |
| *Elephas cypriotes* | 200000 | [20] |
| *Elephas maximus* | 3320691 | [21] |
| *Elephas maximus asurus* | NA | NA |
| *Elephas sp.* | NA | NA |
| *Loxodonta africana* | 4640000 | [5] (page 194) |
| *Loxodonta cyclotis* | 4750000.01 | [2] |
| *Mammut americanum* | 6016667 | [21] (taken as mean of humerus best measurement – to allow for direct comparison of different species) |
| *Mammuthus columbi* | 6630000 | [21] (taken as mean of humerus best measurement – to allow for direct comparison of different species) |
| *Mammuthus primigenius* | 4282889 | [21] (taken as mean of humerus best measurement – to allow for direct comparison of different species) |
| *Phiomia spp* | 1080958 | Molar area [22] using the equation of Bloch *et al.* [1] |
| *Phosphatherium escuilliei* | 15000 | [23] |
| *Moeritherium spp* | 1000000 | [9] |
| *Numidotherium koholense* | 558000 | [23] |
| *Omanitherium dhofarensis* | 624744.3 | Molar area [24] using the equation of Bloch *et al.* [1] |
| *Arcanotherium savagei* | 396016.4 | Molar area [25] using the equation of Bloch *et al.* [1] |
| *Barytherium spp* | 4000000 | [23] |
| *Daouitherium rebouli* | 364000 | [23] |
| *Dugong dugon* | 410000 | [5] (page 208) |
| *Hydrodamalis gigas* | 6738250 | [26] |
| *Prorastomus sirenoides* | 98155.5 | [26] |
| *Protosiren smithae* | 542000 | [26] (taken from *P. fraasi*) |
| *Trichechus inunguis* | 480000 | [26] |
| *Trichechus manatus* | 689185 | [26] |
| *Trichechus senegalensis* | 454000 | [2] |

**14. Fossil ages**

**Table S4.** Minimum and maximum ages of fossil taxa used in the analyses.

| Species | Age range (Ma) | Reference |
| --- | --- | --- |
| *Afrohyrax championi* | 11.6, 20.4 | Miocene [27] |
| *Antilohyrax pectidens* | 28.4, 33.9 | Jebel Qatrani Formation [18]  Early Oligocene [28] |
| *Arcanotherium savagei* | 32.8, 37.2 | Late Eocene [25] |
| *Barytherium spp* | 32.8, 37.2 | Late Eocene [29] |
| *Bunohyrax fajumensis* | 28.4, 33.9 | [30] |
| *Bunohyrax major* | 28.4, 33.9 | [30] |
| *Chambius kasserinensis* | 40.4, 55.8 | Early to Mid-Eocene [31] |
| *Daouitherium rebouli* | 48.6, 55.8 | Ypresian  Gheerbrant *et al.* (2002) |
| *Dilambdogale gheerbranti* | 37 (fixed) | [33] |
| *Dimaitherium patnaiki* | 33.9, 37.2 | Late Eocene [17] |
| *Geniohyus diphycus* | 28.4, 33. | Fayum, Early Oligocene [17] |
| *Herodotius pattersoni* | 28.4, 33.9 | Jebel Qatrani Formation [34]  Early Oligocene [28] |
| *Megalohyrax eocaenus* | 28.4, 33.9 | Jebel Qatrani Formation [35]  Early Oligocene [28] |
| *Megalohyrax* | 28.4, 33.9 | [36] (e.g. *Pliohyrax*) |
| *Metoldobotes* | 28.4, 33.9 | Jebel Qatrani Formation [34]  Early Oligocene [28] |
| *Microhyrax lavocati* | 40.4, 55.8 | Gour Lazib [15]  Early to Middle Eocene [37] |
| *Moeritherium* | 33.9, 48.6 | Eocene [38] |
| *Montanalestes keeblerorum* | 99.7, 112.6 | Aptian-Albian [39] |
| *Myorycteropus africanus* | 11.6, 13.6 | Early Miocene [12] |
| *Numidotherium koholense* | 48.6, 55.8 | Early Eocene [40] |
| *Omanitherium dhofarensis* | 28.1, 33.9 | Early Oligocene [24] |
| *Pachyhyrax crassidentatus* | 28.4, 33.9 | Early Oligocene [41] |
| *Phiomia spp* | 23.02, 37.2 | Late Eocene to Oligocene [42] |
| *Phosphatherium escuilliei* | 48.6, 55.8 | Eocene [43] |
| *Prokennalestes trofimovi* | 99.7, 125.4 | Aptian-Albian [44] |
| *Prorastomus sirenoides* | 40.4, 55.8 | Early Eocene [45] |
| *Protenrec tricuspis* | 15, 23 | Early Miocene [42] |
| *Protosiren smithae* | 33.9, 48.6 | Late Eocene [46] |
| *Saghatherium antiquum* | 28.4, 33.9 | Jebel, Early Oligocene [42] |
| *Saghatherium bowni* | 28.4, 33.9 | Jebel, Early Oligocene [42] |
| *Seggeurius amourensis* | 48.6, 55.8 | Early Eocene [47] |
| *Selenohyrax chatrathi* | 28.4, 33.9 | Jebel, Early Oligocene [48] |
| *Thyrohyrax domorictus* | 28.4, 33.9 | Jebel, Early Oligocene [27] |
| *Thyrohyrax litholagus* | 28.4, 33.9 | Fayum, Early Oligocene [13] |
| *Thyrohyrax meyeri* | 28.4, 33.9 | Fayum, Early Oligocene [13] |
| *Thyrohyrax pygmaeus* | 28.4, 33.9 | Early Oligocene [48] |
| *Titanohyrax andrewsi* | 28.4, 33.9 | Early Oligocene [48] |
| *Titanohyrax angustidens* | 28.4, 33.9 | Fayum, Early Oligocene [13] |
| *Titanohyrax* | 28.4, 33.9 | Fayum, Early Oligocene [13] |
| *Widanelfarasia bowni* | 28.4, 33.9 | Jebel, Early Oligocene [49] |

**15. Data References**

1. Bloch JI, Rose KD, Gingerich PD. 1998. New species of Batodonoides (Lipotyphla, Geolabididae) from the early Eocene of Wyoming: smallest known mammal? J. Mammal. 79, 804-827.

2. Jones KE, *et al.* 2009 Pantheria: A Species-level database of life history, ecology, and geography of extant and recently extinct mammals. Ecology 90, 2648.

3. Kaufman JA, Turner GH, Holroyd PA, Rovero F, Grossman A. 2013 Brain Volume of the Newly-Discovered Species Rhynchocyon udzungwensis (Mammalia: Afrotheria: Macroscelidea): Implications for Encephalization in Sengis. PloS one 8, e58667. (doi: 10.1371/journal.pone.0058667)

4. Goodman SM, Raxworthy CJ, Maminirina CP, Olson LE. 2006 A new species of shrew tenrec (Microgale jobihely) from northern Madagascar. J. Zool. 270, 384-398.

5. Kingdon J, Happold D, Butynski T, Hoffmann M, Happold M, Kalina J. 2013 Mammals of Africa (Vol. 1). A&C Black.

6. Butler PM. 1985 The history of African insectivores. Acta Zool. Fennica. 173, 215-217.

7. Luo ZX, Crompton AW, Sun AL. 2001 A new Mammaliaform from the Early Jurassic and evolution of mammalian characteristics. Science 292, 1535–1540.

8. Seiffert ER. 2010 The oldest and youngest records of afrosoricid placentals from the Fayum Depression of northern Egypt. Acta Palaeontol. Pol. 55, 599-616.

9. Benoit J, Crumpton N, Merigeaud S, Tabuce R. 2014. Petrosal and Bony Labyrinth Morphology Supports Paraphyly of Elephantulus Within Macroscelididae (Mammalia, Afrotheria). J. Mamm. Evol. 21, 173-193.

10. Patterson B. 1965 The fossil elephant shrews (Family Macroscelididae).  Bull. Mus. Comp. Zool., Harvard University 133, 295-335.

11. Tabuce R, *et al.* 2012. New stem elephant‐shrews (Mammalia, Macroscelidea) from the Eocene of Dur At‐Talah, Libya. Palaeontology 55, 945-955.

12. Lehmann T. 2009 Phylogeny and systematics of the Orycteropodidae (Mammalia, Tubulidentata). Zool. J. Linnean Soc. 155: 649-702.

13. Schwartz GT, Rasmussen DT, Smith RJ. 1995 Body-size diversity and community structure of fossil hyracoids. J. Mammal. 76, 1088-1099.

14. Osborn HF. 1906 Milk dentition of the hyracoid Saghatherium from the Upper Eocene of Egypt. Bull. Am. Mus. Nat. Hist 22, 13.

15. Andrews, C. W. 1904. II.—Further Notes on the Mammals of the Eocene of Egypt. Geological Magazine (Decade V) 1, 157-162.

16. Damuth JD, MacFadden BJ. (Eds.). 1990. Body size in mammalian paleobiology: estimation and biological implications. Cambridge University Press.

17. Barrow E, Seiffert ER, Simons EL. 2010 A primitive hyracoid (Mammalia, Paenungulata) from the early Priabonian (late Eocene) of Egypt. J. Syst. Palaeontol. 8, 213-244.

18. Rasmussen DT, Simons EL. 2000 Ecomorphological diversity among Paleogene hyracoids (Mammalia): a new cursorial browser from the Fayum, Egypt. J. Vert. Paleontol. 20, 167-176.

19. Raia P, Barbera C, Conte M. 2003 The fast life of a dwarfed giant. Evol. Ecol. 17, 293-312.

20. Davies P, Lister A. 2001 Palaeloxodon cypriotes, the dwarf elephant of Cyprus: size and scaling comparisons with P. falconeri (Sicily–Malta) and mainland P. antiquus. Proceedings of the First International Congress “The World of Elephants”, Rome, pp. 479–480.

21. Christiansen P. 2004. Body size in proboscideans, with notes on elephant metabolism. Zool. J. Linn. Soc. 140, 523-549.

22. Sanders WJ, Kappelman J, Rasmussen DT. 2004 New large-bodied mammals from the late Oligocene site of Chilga, Ethiopia. Acta Palaeontol. Pol. 49, 365-392.

23. Saarinen JJ, *et al.* 2014 Patterns of maximum body size evolution in Cenozoic land mammals: eco-evolutionary processes and abiotic forcing. Proc. R. Soc. B: Biol. Sci.  281. (doi: http://dx.doi.org/10.1098/rspb.2013.2049)

24. Seiffert ER, Nasir S, Al-Harthy A, Groenke JR, Kraatz BP, Stevens NJ, Al-Sayigh AR. 2012 Diversity in the later Paleogene proboscidean radiation: a small barytheriid from the Oligocene of Dhofar Governorate, Sultanate of Oman. Naturwissenschaften 99,133-141.

25. Court N. 1995 A new species of Numidotherium (Mammalia: Proboscidea) from the Eocene of Libya and the early phylogeny of the Proboscidea. J. Vert. Paleontol. 15, 650-671.

26. Benoit J, Adnet S, El Mabrouk E, Khayati H, Ali MBH, Marivaux L, Marzeraud G, Merigeaud S, Vianey-Liaud M, Tabuce R. 2013 Cranial remain from Tunisia provides new clues for the origin and evolution of Sirenia (Mammalia, Afrotheria) in Africa. PloS one 8, 54307. (doi 0.1371/journal.pone.0054307)

27. Barrow EC, Seiffert ER, Simons EL. 2012 Cranial morphology of Thyrohyrax domorictus (Mammalia, Hyracoidea) from the early Oligocene of Egypt. J. Vert. Paleontol. 32, 166-179.

28. Seiffert, ER. 2006. Revised age estimates for the later Paleogene mammal faunas of Egypt and Oman. Proc. Natl. Acad. Sci. USA 103:5000-5005.

29. Andrews, CW. 1901. Über das Vorkommen von Proboscidiern in unter− tertiären Ablagerungen Aegyptens. Tageblatt des V Internationalen Zoologischen Kongresses, Berlin 6: 4–5.

30. Tabuce, R., Coiffait, B., Coiffait, PE., Mahboubi, M., and Jaeger, JJ. 2000. A new species of Bunohyrax (Hyracoidea, Mammalia) from the Eocene of Bir el Ater (Algeria). Comptes Rendus de l'Académie des Sciences-Series IIA-Earth and Planetary Science 331:61-66.

31. Tabuce R, Coiffait B, Coiffait PE, Mahboubi M, and Jaeger JJ. 2001. A new genus of Macroscelidea (Mammalia) from the Eocene of Algeria: a possible origin for elephant-shrews. J. Vert. Paleontol. 21: 535-546.

32. Gheerbrant E, Sudre J, Cappetta H, Iarochéne M, Amaghzaz M, and Bouya B. 2002. A new large mammal from the Ypresian of Morocco: Evidence of surprising diversity of early proboscideans. Acta Palaeontol Pol. 47:493-506.

33. Seiffert ER. 2010 The oldest and youngest records of afrosoricid placentals from the Fayum Depression of northern Egypt. Acta Palaeontol. Pol. 55, 599-616.

34. Simons, EL, Holroyd, PA, and Bown, TM. 1991. Early tertiary elephant-shrews from Egypt and the origin of the Macroscelidea. Proc. Natl. Acad. Sci. USA 88:9734-9737.

35. Thewissen, JGM and Simons, EL. 2001. Skull of Megalohyrax eocaenus (Hyracoidea, mammalia) from the Oligocene of Egypt. J. Vert. Paleontol. 21: 98-106.

36. Andrews, CW. 1903. I.—Notes on an Expedition to the Fayûm, Egypt, with Descriptions of Some New Mammals. Geological Magazine (Decade IV) 10:337-343.

37. Solé, F, Lhuillier, J, Adaci, M, Bensalah, M, Mahboubi, MH, and Tabuce, R. 2014. The hyaenodontidans from the Gour Lazib area (? Early Eocene, Algeria): implications concerning the systematics and the origin of the Hyainailourinae and Teratodontinae. J. Syst. Palaeontol. 12:303-322.

38. Delmer C, Mahboubi M, Tabuce R., and Tassy P. 2006. A new species of Moeritherium (Proboscidea, Mammalia) from the Eocene of Algeria: new perspectives on the ancestral morphotype of the genus. Palaeontology 49:421-434.

39. Cifelli, R. L. 1999. Tribosphenic mammal from the North American Early Cretaceous. Nature 401:363-366.

40. Mahboubi M, Ameur R, Crochet J-Y,  Jaeger JJ. 1984. Earliest known prosboscidean from Early Eocene of north-west Africa. Nature 308:543–544.

41. Kappelman J, *et al.* 2003. Oligocene mammals from Ethiopia and faunal exchange between Afro-Arabia and Eurasia. Nature 426:549-552.#

42. Tabuce R, Asher RJ, Lehmann T. 2008 Afrotherian mammals: a review of current data. Mammalia 72, 2-14.

43. Gheerbrant E. 2009. Paleocene emergence of elephant relatives and the rapid radiation of African ungulates. Proc. Natl. Acad. Sci. USA 106:10717-10721.

44. Wible, J. R., Rougier, G. W., Novacek, M. J., & McKENNA, M. C. (2001). Earliest eutherian ear region: a petrosal referred to Prokennalestes from the Early Cretaceous of Mongolia. American Museum Novitates, 1-44.

45. Savage RJG, Domning DP, and Thewissen JGM. 1994. Fossil Sirenia of the West Atlantic and Caribbean region. V. The most primitive known sirenian, Prorastomus sirenoides Owen, 1855. J.Vert. Paleontol. 14:427-449.

46. Domning DP, and Gingerich PD. 1994. Protosiren smithae, new species (Mammalia, Sirenia), from the late Middle Eocene of Wadi Hitan, Egypt. Contr. Mus. Paleontol. Univ. Michigan 29, 69-87.

47. Court N, and Mahboubi, M. 1993. Reassessment of lower Eocene Seggeurius amourensis: aspects of primitive dental morphology in the mammalian order Hyracoidea. J. Paleo. 67:889-893.

48. Rasmussen DT, and Simons EL. 1988. New Oligocene hyracoids from Egypt. J. Vert. Paleontol. 8:67-83.

49. Seiffert ER., and Simons EL. 2000. Widanelfarasia, a diminutive placental from the late Eocene of Egypt. Proc. Natl. Acad. Sci. USA 97:2646-2651.
